# RpoN is required for the motility and contributes to the killing ability of *Plesiomonas shigelloides*

**DOI:** 10.1186/s12866-022-02722-8

**Published:** 2022-12-12

**Authors:** Junxiang Yan, Xueqian Guo, Jinghao Li, Yuehua Li, Hongmin Sun, Ang Li, Boyang Cao

**Affiliations:** 1 TEDA Institute of Biological Sciences and Biotechnology, Nankai University, No. 23, Hongda StreetTianjin Economic and Technological Development Area, Tianjin, 300457 China; 2 Key Laboratory of Molecular Microbiology and Technology of the Ministry of Education, Nankai University, No.23, Hongda StreetTianjin Economic and Technological Development Area, Tianjin, 300457 China; 3 Tianjin Key Laboratory of Microbial Functional Genomics, TEDA College, Nankai University, No.23, Hongda StreetTianjin Economic and Technological Development Area, Tianjin, 300457 China; 4 State Key Laboratory of Medicinal Chemical Biology, College of Pharmacy and Tianjin Key Laboratory of Molecular Drug Research, Nankai University, Haihe Education Park, 38 Tongyan Road, Tianjin, 300353 China; 5 TEDA Institute of Biological Sciences and Biotechnology, Nankai University, No.23, Hongda StreetTianjin Economic and Technological Development Area, Tianjin, 300457 China; 6 Key Laboratory of Molecular Microbiology and Technology of the Ministry of Education, Nankai University, No. 23, Hongda StreetTianjin Economic and Technological Development Area, Tianjin, 300457 China; 7 Tianjin Key Laboratory of Microbial Functional Genomics, TEDA College, Nankai University, No. 23, Hongda StreetTianjin Economic and Technological Development Area, Tianjin, 300457 China

**Keywords:** *Plesiomonas shigelloides*, RpoN, RNA sequencing, Motility, Killing ability, T6SS, T2SS-2

## Abstract

**Background:**

RpoN, also known as σ^54^, first reported in *Escherichia coli*, is a subunit of RNA polymerase that strictly controls the expression of different genes by identifying specific promoter elements. RpoN has an important regulatory function in carbon and nitrogen metabolism and participates in the regulation of flagellar synthesis, bacterial motility and virulence. However, little is known about the effect of RpoN in *Plesiomonas shigelloides.*

**Results:**

To identify pathways controlled by RpoN, RNA sequencing (RNA-Seq) of the WT and the *rpoN* deletion strain was carried out for comparison. The RNA-seq results showed that RpoN regulates ~ 13.2% of the *P. shigelloides* transcriptome, involves amino acid transport and metabolism, glycerophospholipid metabolism, pantothenate and CoA biosynthesis, ribosome biosynthesis, flagellar assembly and bacterial secretion system. Furthermore, we verified the results of RNA-seq using quantitative real-time reverse transcription PCR, which indicated that the absence of *rpoN* caused downregulation of more than half of the polar and lateral flagella genes in *P. shigelloides*, and the Δ*rpoN* mutant was also non-motile and lacked flagella. In the present study, the ability of the Δ*rpoN* mutant to kill *E. coli* MG1655 was reduced by 54.6% compared with that of the WT, which was consistent with results in RNA-seq, which showed that the type II secretion system (T2SS-2) genes and the type VI secretion system (T6SS) genes were repressed. By contrast, the expression of type III secretion system genes was largely unchanged in the Δ*rpoN* mutant transcriptome and the ability of the Δ*rpoN* mutant to infect Caco-2 cells was also not significantly different compared with the WT.

**Conclusions:**

We showed that RpoN is required for the motility and contributes to the killing ability of *P. shigelloides* and positively regulates the T6SS and T2SS-2 genes.

**Supplementary Information:**

The online version contains supplementary material available at 10.1186/s12866-022-02722-8.

## Background

After many years in the family *Vibrionaceae*, the genus *Plesiomonas*, represented by a single species, *Plesiomonas shigelloides*, currently resides in the family *Enterobacteriaceae* [1]. *P. shigelloides* is a gram-negative opportunistic pathogen that causes acute secretory gastroenteritis, an invasive shigellosis-like disease, and a cholera-like illness [2–4]. Extra intestinal infections are also associated with *P. shigelloides*, such as bacteremia, pseudoappendicitis and meningitis [5–7]. RpoN (σ^54^) is widely found in pathogenic bacteria. It binds to the core RNA polymerase and regulates the transcription of many functional genes in an enhancer-binding protein (EBP)-dependent manner, in general, bacteria contain one or two RpoNs but multiple EBPs [8]. Regulation of RpoN has been extensively studied in many bacterias. In *Escherichia coli*, RpoN affects the nitrogen and carbon metabolism, fermentation, cell envelope biogenesis, stress fitness, and pathogenesis [9, 10]. The RpoN regulon is very diverse, controlling genes involved in the response to nitrogen limitation, nitric oxide stress, availability of alternative carbon sources, toxic levels of zinc and nucleic acid damage in *Salmonella typhimurium* [11]. In *Pseudomonas putida*, RpoN affects the utilization of nitrate, urea, and uncharged amino acids as nitrogen sources, as well as lysine, C4-dicarboxylates, and alpha-ketoglutarate as carbon sources [12, 13]. RpoN also regulates the susceptibility to tobramycin [14], quinolones, and carbapenems [15, 16] in *P. aeruginosa*. In addition, RpoN controls bacterial exopolysaccharide production and biofilm formation [17, 18], quorum sensing [19, 20], environmental adaptation [21, 22], and antibiotic resistance [23, 24] in other bacteria.

*P. shigelloides* is a unique member of the Enterobacteriaceae family that contains two different gene clusters, one exclusively for lateral flagella biosynthesis and the other one containing the biosynthetic polar flagella genes [25]. However, the transcriptional regulatory network of polar and lateral flagella is currently unreported. RpoN is known to be required for bacterial motility and flagellar synthesis, such as in *V. cholerae* [26–28], *Vibrio parahaemolyticus* [29], *Vibrio campbellii* [30], *Aeromonas hydrophila* [31, 32], *Pseudomonas aeruginosa* [33, 34], *Caulobacter crescentus* [35], *Helicobacter pylori* [36] and *Rhodobacter sphaeroides* [37]. In *E. coli*, σ^70^ is mainly involved in the synthesis and regulation of flagella; however, recent studies have also reported that σ^54^ also has an important regulatory role on the motility [38, 39].

The Type VI secretion system is a protein translocation nanomachine that is widespread among gram-negative bacteria and is also a contact-dependent bacterial weapon that allows for the direct killing of competitors through the translocation of proteinaceous toxins [40, 41]. These proteinaceous toxins have a wide variety of functions within target cells that ultimately help the secreting cell gain a competitive advantage [42]. The primary role of the T6SS appears to be to act against competitor bacteria. T6SS genes are distributed over the *P. shigelloides* chromosome and consist of two clusters: a large cluster and an auxiliary clusters. The large cluster encodes the majority of the structural T6SS components, including the outer sheath proteins, key proteins for the tip of the T6SS and proteins that assemble at the inner and outer membranes [43]. Additionally, the large cluster encodes a gene necessary for disassembly of the T6SS, *clpV*, and an essential transcriptional regulator, *vasH* [44, 45]. VasH is a σ^54^-dependent transcription factor encoded in the large T6SS cluster that positively regulates the two auxiliary clusters essential for T6SS activity [46].

The T2SS is a multi-protein complex used by many gram-negative bacteria to move substrates across their cell membrane [47]. It is a key virulence factor in many human pathogens including *Acinetobacter baumannii* [48], *Klebsiella pneumoniae* [49], *Pseudomonas aeruginosa* [50], *Vibrio cholerae* [51] and enterotoxigenic *Escherichia coli* [52]. Many diverse effectors and toxins depend on the T2SS for secretion [53–55], such substrates are involved in adhesion, biofilm formation, nutrient acquisition, colonization, and invasion [56]. The canonical T2SS operon contains approximately 13 genes, often arranged in a single operon, named *gspC* to *gspO* [57, 58]. In the present study, we also found a certain association between RpoN and the type II secretion system.

The effects of RpoN in *P. shigelloides* are unknown; therefore, we used *P. shigelloides* as the research object and revealed the function of RpoN in *P. shigelloides*.

## Results

### Phylogenetic analysis of RpoN

**Fig. 1:**
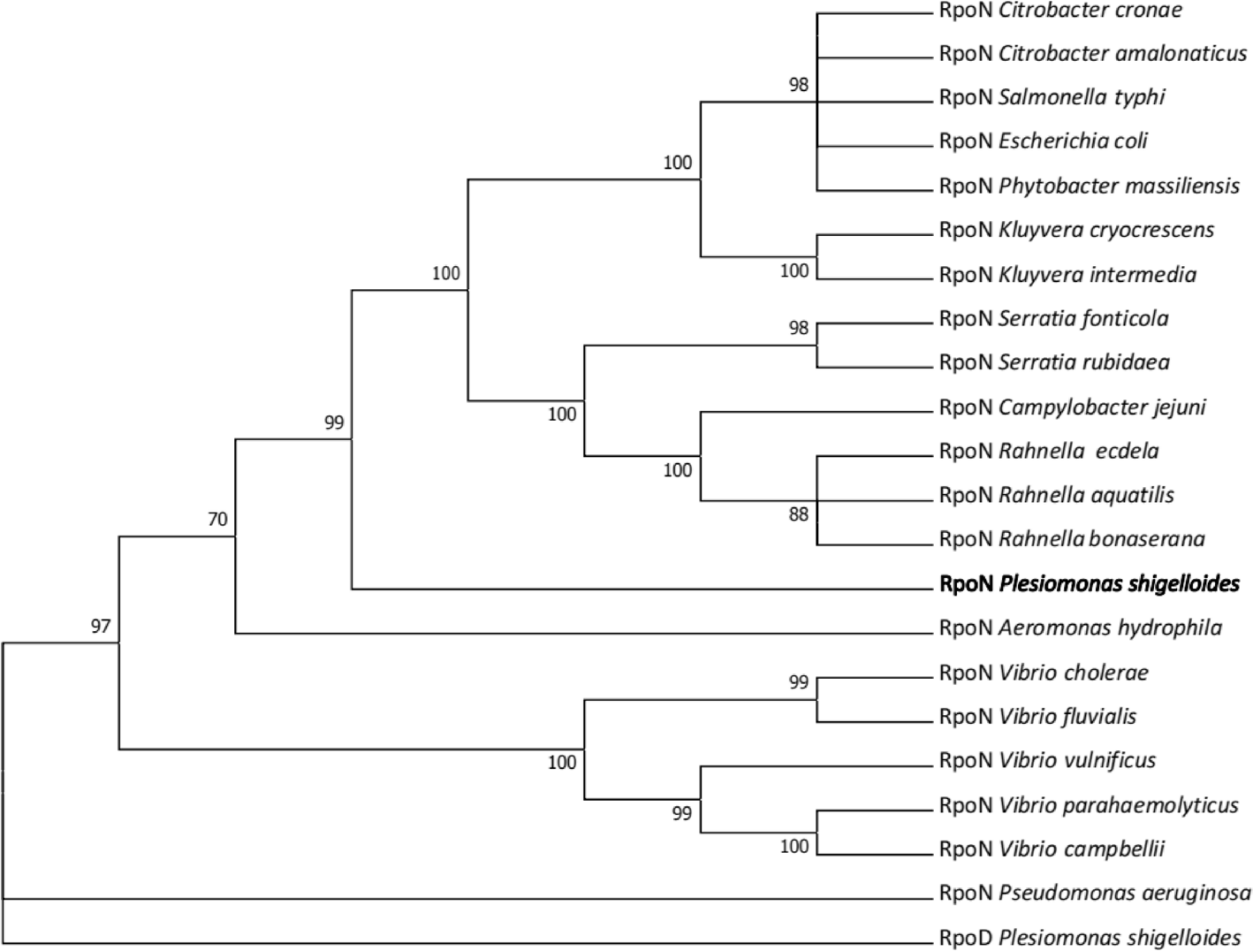
Phylogenetic analysis of RpoN. An unrooted phylogenetic tree constructed using the neighbor joining method based on RpoN amino acid sequences, Bootstrap values were based on 1000 replications and only values greater than 70% are shown. All amino acid sequences were downloaded from the National Center for Biotechnology Information

To analyze the function of the RpoN, a phylogenetic tree based on the RpoN amino acid sequences was constructed and the RopD protein of *P. shigelloides* was selected as the outgroup control (Fig. 1). The neighbor-joining tree consisting of 21 species of bacteria, RpoN of *P. shigelloides* was closer to *Escherichia coli* and *Salmonella typhi* than *Vibrio*. Moreover, RpoN of *P. aeruginosa* formed a separate evolutionary branch.

### Transcriptome sequencing revealed gene expression related to RpoN of the *P. shigelloides*

**Fig. 2:**
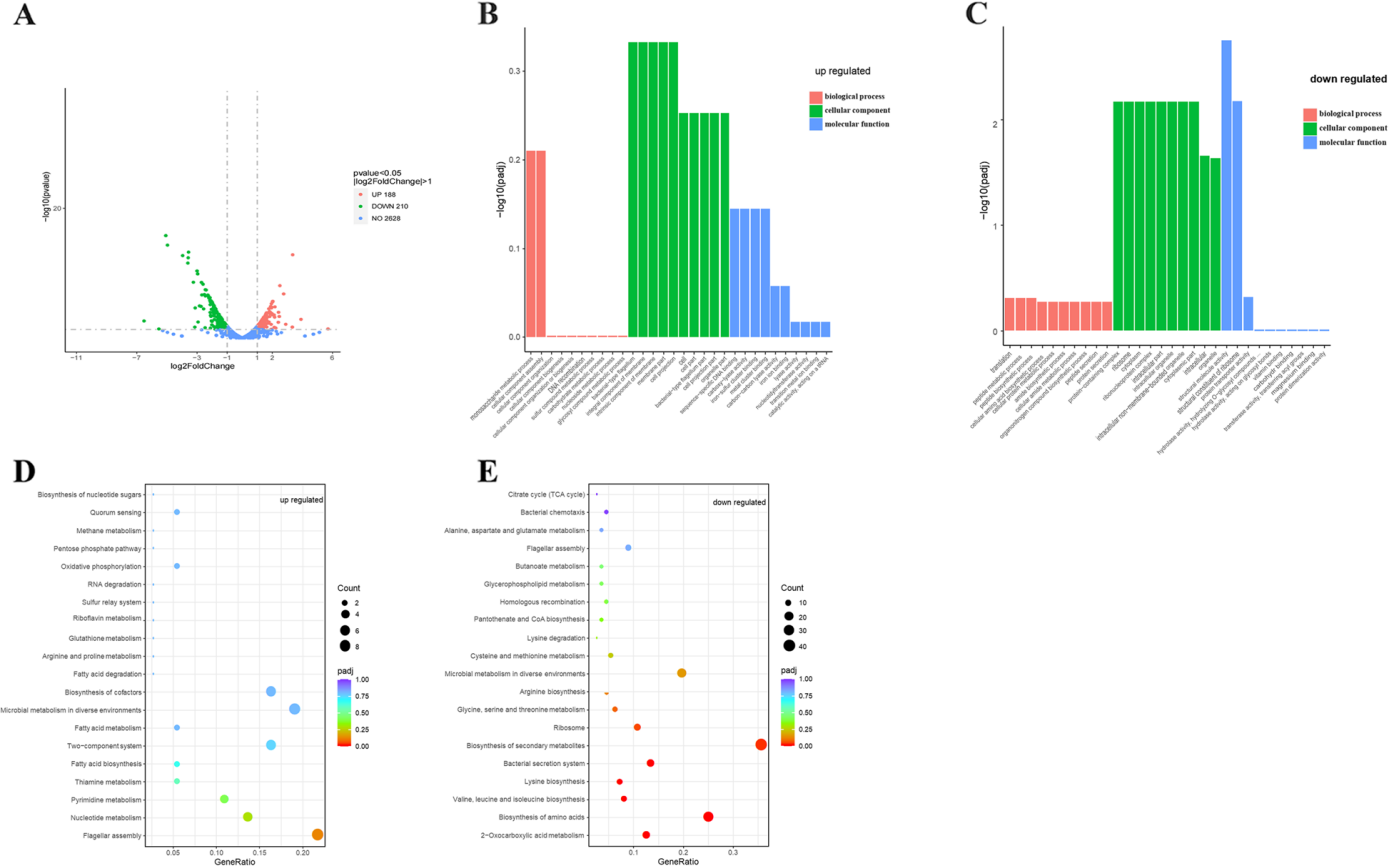
Transcriptomic analysis of the *P. shigelloides* between WT and Δ*rpoN* strains. **A** The volcano plot of differential expressed genes (DEGs), red circle was up- regulated genes, green circle was down-regulated genes, blue circle was no DEGs; **B** GO enrichment of up-regulated DEGs; **C** GO enrichment of down-regulated DEGs, The enrichment analysis of GO function is divided into three major categories: Biological process and Cellular component and Molecular function. **D** KEGG enrichment of up-regulated DEGs; **E** KEGG enrichment of down-regulated DEGs, the GeneRatio refers to the ratio of the number of DEGs in the pathway and the number of all annotated genes in the pathway

To investigate the regulatory role of RpoN in *P. shigelloides*, transcriptome profiles of the WT and Δ*rpoN* strains were analyzed using RNA-seq. The RNA-seq results showed that RpoN regulates approximately 13.2% of the *P. shigelloides* transcriptome: a total of 398 DEGs in the Δ*rpoN* strain were identified in comparison with the WT strain, including 210 downregulated genes and 188 upregulated genes (Fig. 2A, Table S1A). GO enrichment analysis of the DEGs showed that they were mainly classified as Cellular component, followed by Molecular function, and finally by Biological process (Fig. 2B and C). KEGG signaling pathway analysis showed that the upregulated genes were involved in flagellar assembly, microbial metabolism in diverse environments, biosynthesis of cofactors, two-component system, nucleotide metabolism, pyrimidine metabolism, quorum sensing and oxidative phosphorylation (Fig. 2D). The downregulated genes were involved in biosynthesis of secondary metabolites, biosynthesis and metabolism of amino acids, the bacterial secretion system, 2 − Oxocarboxylic acid metabolism, flagellar assembly, bacterial chemotaxis, pantothenate and CoA biosynthesis, butanoate and glycerophospholipid metabolism (Fig. 2E). Among the upregulated DEGs were genes responsible for the biosynthesis of cofactors (*iscS*, *pyrF*, *pdxJ*, *yjjG*, *thiH*), nucleotide metabolism (*guaA*, *guaB*, *pyrH*, *tdk*), and oxidative phosphorylation (*ppk2*, *cydB*). Among the downregulated DEGs were genes responsible for biosynthesis and metabolism of amino acids (*argA/B/C/D/H*, *leuA/B/C/D*, *thrA/B/C*, *metL*, *cysE*, *lysC*, *trpD*, *hisD*), glycerophospho-lipid metabolism (*glpA/C/D/Q/T)*, pantothenate and CoA biosynthesis (*ilvD/E/G/M*), and butanoate metabolism (*phbB*, *putA*).

### RpoN influenced the motility, flagellar synthesis, motor assembly and growth of *P. shigelloides*

**Fig. 3:**
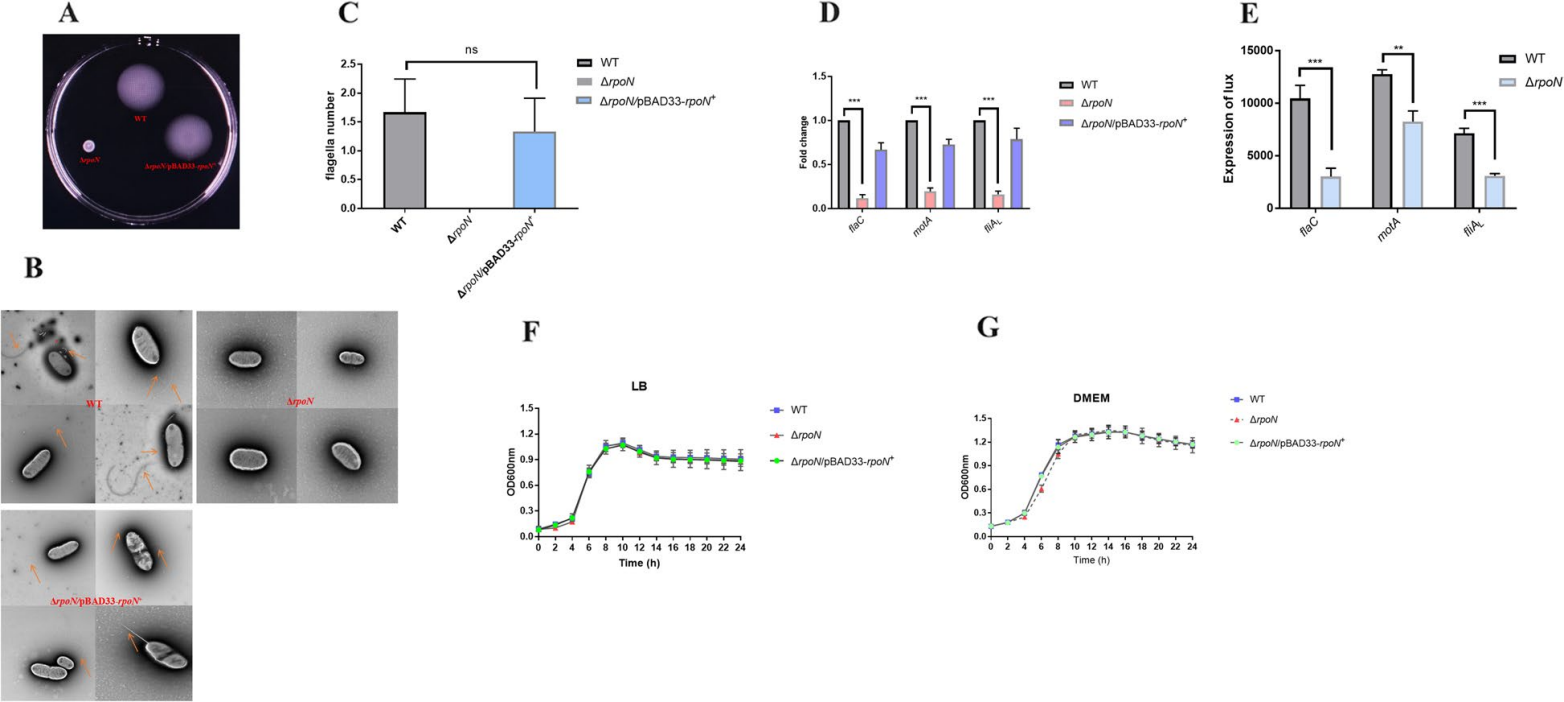
RpoN is essential for the motility of *P. shigelloides* and affects the growth of *P*. *shigelloides*. **A** The motility of WT, Δ*rpoN* and Δ*rpoN/*pBAD33-*rpoN*^+^ strains grown in swimming agar plate. **B** TEM visualization of the flagella produced by the WT, Δ*rpoN* and Δ*rpoN/*pBAD33-*rpoN*^+^ strains. The hollow bacterial flagella were pointed by the colored arrows. **C** The picture is drawn based on the observed difference in the number of all flagella produced by WT, Δ*rpoN* and Δ*rpoN/*pBAD33-*rpoN*^+^. **D** Transcription verification of *flaC*, *motA* and *fliA*_L_ by qRT-PCR. **E** Expression of *flaC*-lux, *motA*-lux, and *fliA*_L_-lux in the WT and Δ*rpoN* mutant. **F** The differences in growth between the WT, Δ*rpoN* mutant and Δ*rpoN*/pBAD33-*rpoN*^+^ in LB liquid medium, and the optical density at 600 nm (OD_600_) was monitored. **G** The differences in growth between the WT, Δ*rpoN* mutant and Δ*rpoN*/pBAD33-*rpoN*.^+^ in DMEM, and the optical density at 600 nm (OD_600_) was monitored. Significant differences were indicated by asterisks (*** *p* ≤ .001; ** *p* ≤ .01; * *p* ≤ .05)

In this study, the effect of RpoN on the motility of *P. shigelloides* was validated by constructing the Δ*rpoN* mutant and Δ*rpoN/*pBAD33-*rpoN*^+^ to observe their migration in swimming agar plates. The swimming agar plate results showed that the motility of Δ*rpoN* mutant was basically lost; however, the motility of the Δ*rpoN/*pBAD33-*rpoN*^+^ complementation strain was restored to the WT level. Thus, RpoN positively controls the motility of *P. shigelloides* (Fig. 3A). Meanwhile, the flagella produced by the WT, Δ*rpoN*, and Δ*rpoN/*pBAD33-*rpoN*^+^ strains were observed using TEM, which indicated that a lack of RpoN influences the flagellar synthesis and assembly in *P. shigelloides*, consistent with the motility assay (Fig. 3B and C). In our RNA-seq analysis, we found that over half of all polar and lateral flagella genes had significantly lower expression in the Δ*rpoN* mutant relative to the WT (Fig. S1A and S1B). Subsequently three greatest downregulated genes, *flaC* and *fliA*_L_ for flagellar synthesis and *motA* for motor assembly, were selected for validation using qRT-PCR in the WT, Δ*rpoN*, and Δ*rpoN/*pBAD33-*rpoN*^+^ strains (Fig. 3D). The results of qRT-PCR were consistent with RNA-seq analysis, suggesting the effects of RpoN on motility gene expression could be caused by RpoN-mediated regulation. Given the significant downregulation of *flaC*, *motA*, and *fliA*_L_ in the RNA-seq data, we further validate the effect of RpoN on the three genes by constructing *flaC*, *motA* and *fliA*_L_ promoter-lux fusion in the Δ*rpoN* mutant and WT strains. The expression levels of *flaC*, *motA*, and *fliA*_L_ promoter-*lux* fusions in the Δ*rpoN* mutant were lower than those in the WT (Fig. 3E). In addition, the differences in growth between the WT and Δ*rpoN* mutant in LB liquid medium and DMEM were compared. When grown in LB liquid, the growth of the Δ*rpoN* mutant slightly lagged behind that of the WT in the lag phase (Fig. 3F). When grown in DMEM, the growth of Δ*rpoN* mutant lagged behind that of the WT in the log phase (Fig. 3G). Growth of the *rpoN* complementation strain completely restored to the WT level indicated that growth metabolism genes of *P. shigelloides* in the lag and log phase were regulated by RpoN, which is in line with the RNA-seq results that RpoN plays an important regulatory function in carbon and nitrogen metabolism in *P. shigelloides*.

### RpoN contributes to the killing ability in *P. shigelloides* by positively regulating the expression of T6SS clusters

**Fig. 4:**
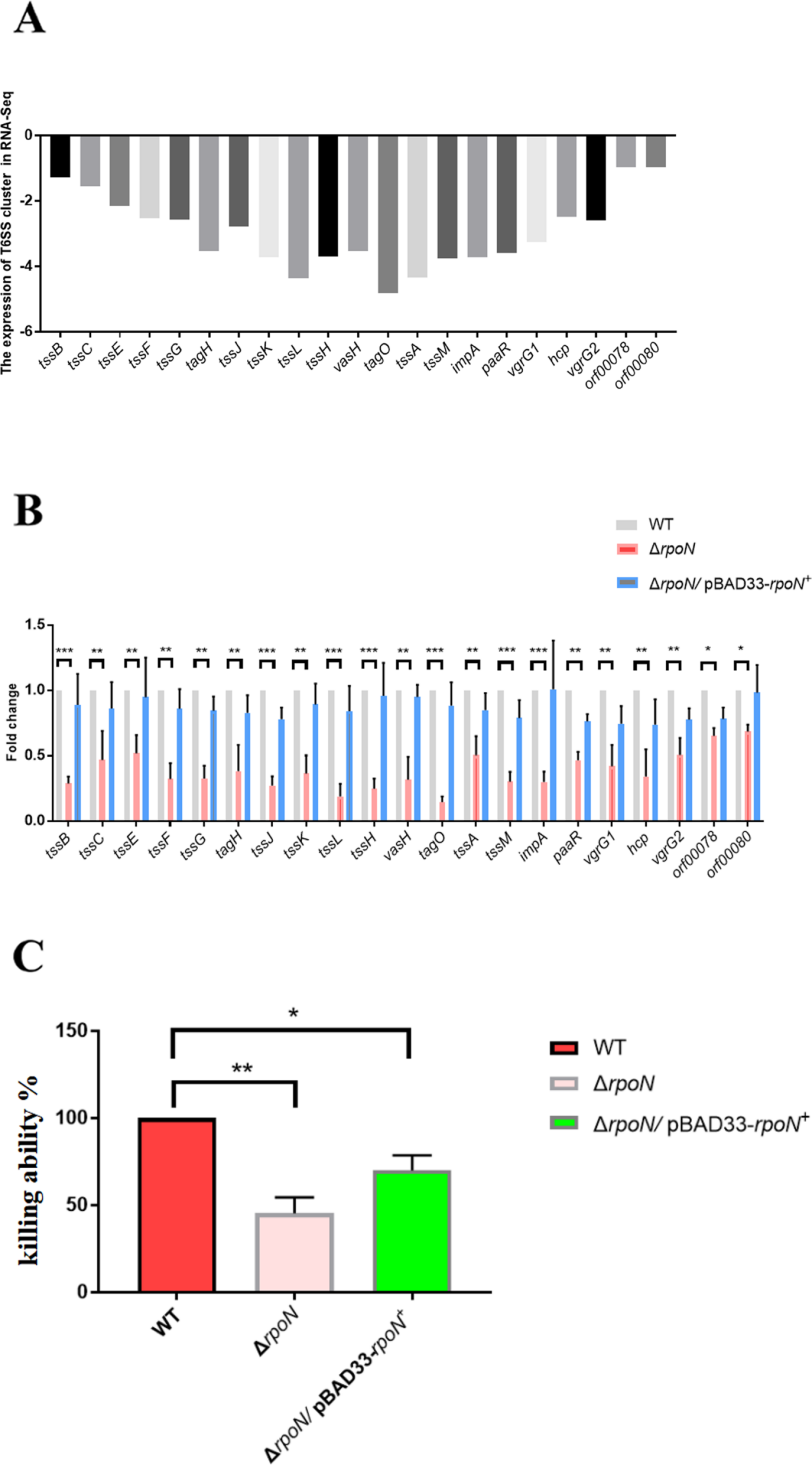
RpoN is involved in killing *E.coli* MG1655 by positively regulating the expression of T6SS clusters. A RNA-seq analysis of the transcription levels of T6SS clusters. **B** Transcription verification of T6SS clusters in the WT, Δ*rpoN* and Δ*rpoN/*pBAD33-*rpoN*^+^ by qRT-PCR. **C** Killing assay of the WT, Δ*rpoN* and Δ*rpoN*/pBAD33-*rpoN*^+^. Killing ability of Δ*rpoN* and Δ*rpoN*/pBAD33-*rpoN*.^+^ were reported as percentage relative to the WT. Significant differences were indicated by asterisks (*** *p* ≤ .001; ** *p* ≤ .01; * *p* ≤ .05)

The T6SS protects *P. shigelloides* by directly translocating toxins to its neighboring bacteria and secreting virulence effectors into the host cells. The T6SS machinery contains two parts, a bacteriophage-like structure and a membrane anchor, to penetrate target cell membranes and secrete effector proteins. Here, transcriptomic profiling of the Δ*rpoN* mutant revealed a positive regulatory role of RpoN on both the large cluster and auxiliary cluster of the T6SS (Fig. 4A). At the same time, transcription of the two clusters of T6SS was verified using qRT-PCR, the results of which were consistent with those of the RNA-seq analysis (Fig. 4B). Subsequently, the killing assay was used to compare the ability of the WT, Δ*rpoN*, and Δ*rpoN*/pBAD33-*rpoN*^+^ strains to kill *E. coli* MG1655, with the aim of verifying that RpoN is required for the killing ability in *P. shigelloides* by positively regulating the expression of T6SS clusters. Compared with the WT, the Δ*rpoN* mutant showed a 54.6% reduction in its ability to kill MG1655, whereas the Δ*rpoN*/pBAD33-*rpoN*^+^ complementation strain could restore the killing ability partially, reaching only about 70% levels compared with that of the WT (Fig. 4C). Hemolysin Co-Regulated Protein (Hcp) is a key effector protein, and the normal expression and secretion of Hcp is a sign of T6SS function. Interestingly, we found RpoN in the DNA pull-down assay previously carried out for the *hcp* gene (Table S1B), suggesting that RpoN could directly regulate *hcp* expression.

### RpoN positively regulates the T2SS-2 cluster associated with the killing ability of *P. shigelloides*

**Fig. 5:**
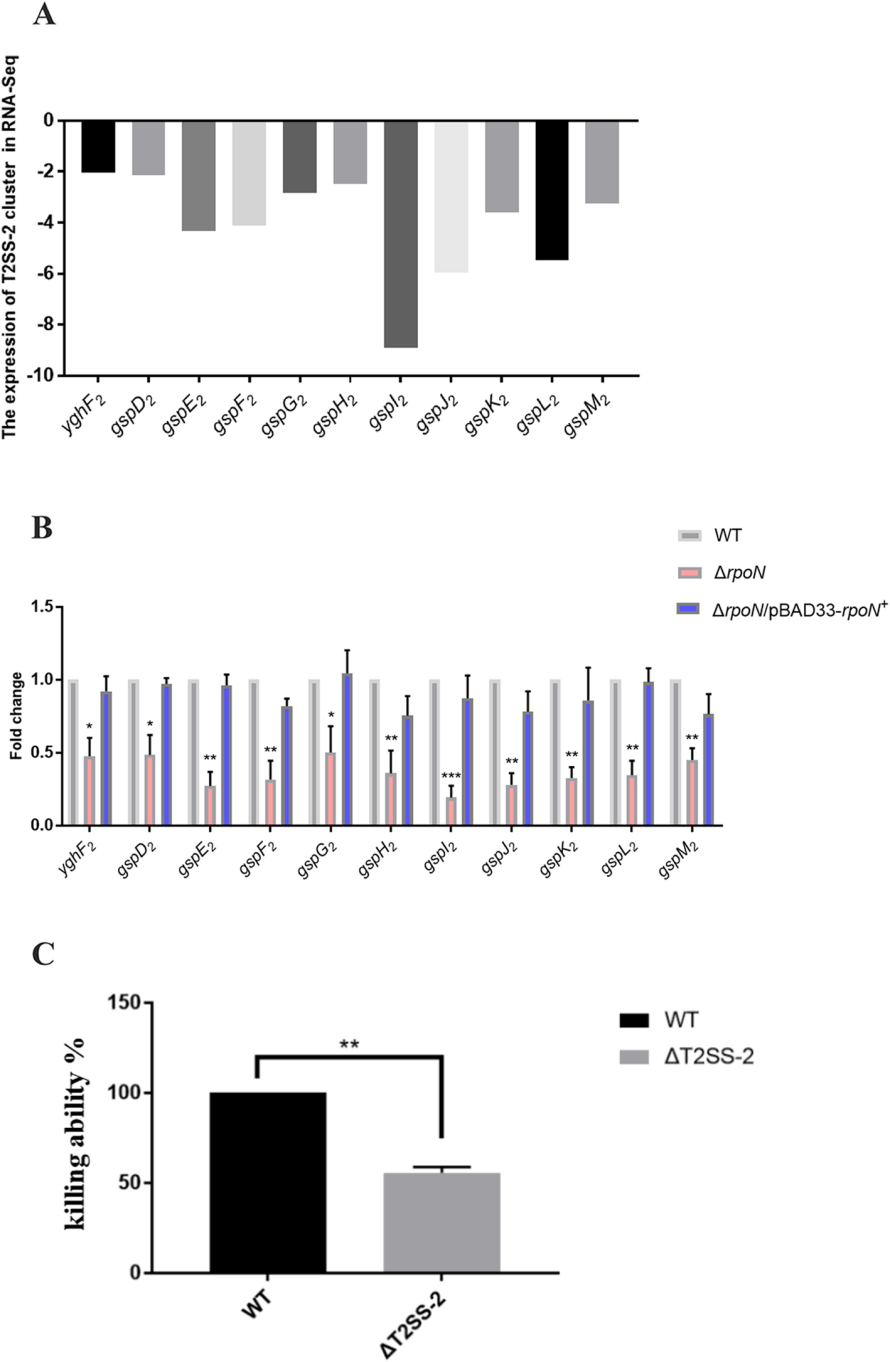
RpoN positively regulates the T2SS-2 cluster associated with the killing ability of *P. shigelloides*. **A** RNA-seq analysis of the transcription levels of T2SS-2 cluster. (**B**) Transcription verification of T2SS-2 cluster in the WT, Δ*rpoN* and Δ*rpoN/*pBAD33-*rpoN*.^+^ by qRT-PCR. **C** Killing assay of the WT and ΔT2SS-2. Killing ability of ΔT2SS-2 was reported as percentage relative to the WT. Significant differences were indicated by asterisks (*** *p* ≤ .001; ** *p* ≤ .01; * *p* ≤ .05)

In addition to the differential expression of the two gene clusters of T6SS in the transcriptomic profile of the Δ*rpoN* mutant, we also identified another secretion system, T2SS-2, which is positively regulated by RpoN. Many diverse effectors and toxins involved in adhesion, biofilm formation, nutrient acquisition, colonization, and invasion, depend on the T2SS for secretion. We found that two types of T2SS were distributed in *P. shigelloides* genomes: The T2SS-1 and T2SS-2 clusters consisted of 12 and 11 genes, respectively. RNA-seq analysis of the transcription levels of the T2SS-2 cluster is shown in Fig. 5A and the qRT-PCR verification of the transcription of the T2SS-2 cluster in the WT, Δ*rpoN*, and Δ*rpoN/*pBAD33-*rpoN*^+^ is shown in Fig. 5B. The results indicated that RpoN positively regulates the T2SS-2 cluster in *P. shigelloides*. However, the expression of the T2SS-1 cluster in the transcriptomic profiling of the Δ*rpoN* mutant was not significantly different (Fig. S1C). Next, we deleted the T2SS-2 cluster to verify whether it was associated to the killing ability of *P. shigelloides*. The killing assay indicated that deletion of the T2SS-2 cluster reduced the ability of *P. shigelloides* to kill *E. coli* MG1655 (Fig. 5C). Combined with the differential expression of the T2SS-2 cluster in the RNA-seq analysis, verified by qRT-PCR, we hypothesized that RpoN positively regulates the T2SS-2 cluster, which is associated with the killing ability of *P. shigelloides*.

### The overall regulation of T3SS cluster expression by RpoN showed no significant difference, and RpoN had little effect in the ability of *P. shigelloides* to infect Caco-2 cells

**Fig. 6:**
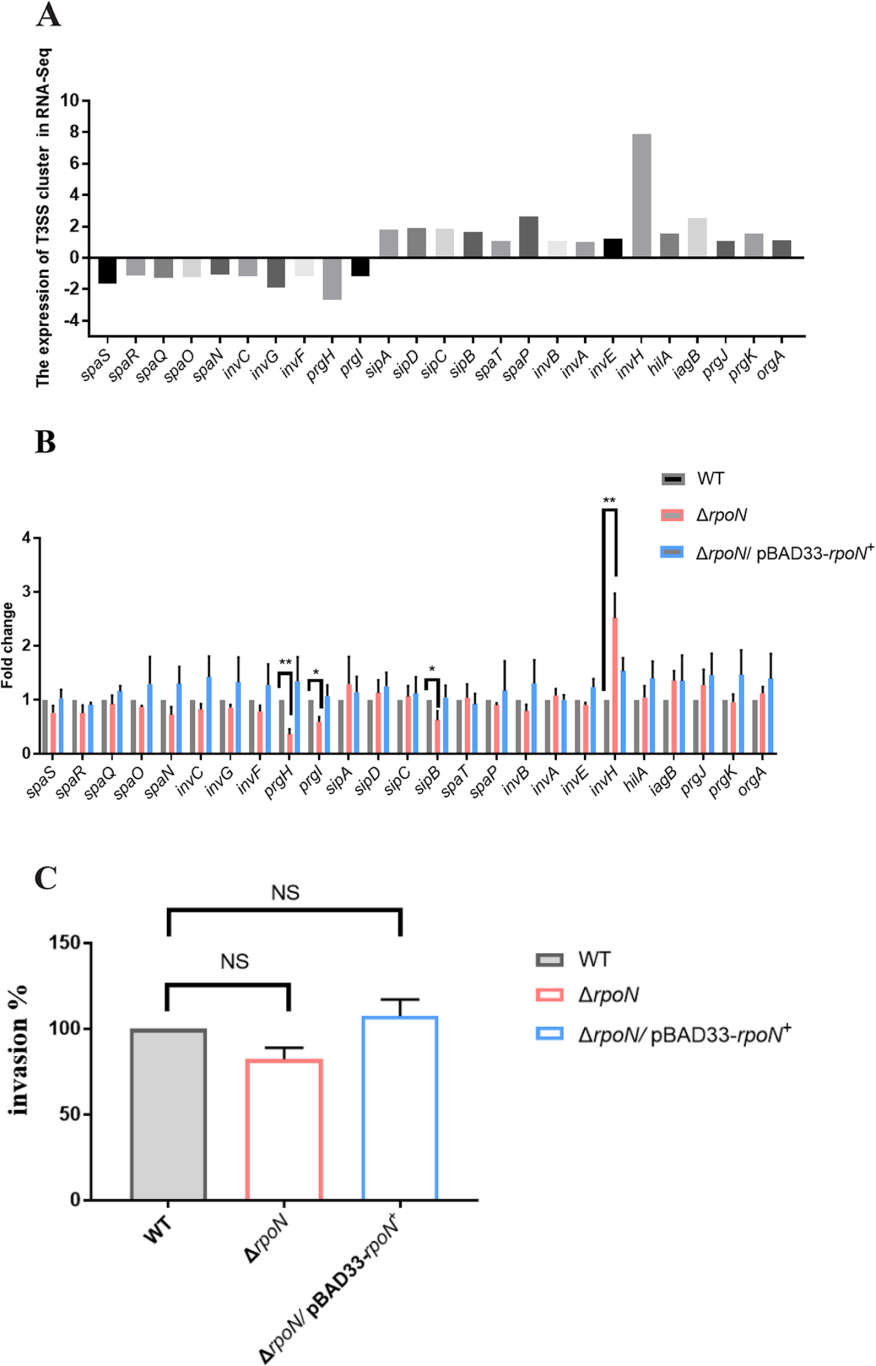
RpoN showed no significant difference in the ability of *P. shigelloides* to infect Caco-2 cells and regulate the T3SS cluster expression. **A** RNA-seq analysis of the transcription levels of T3SS cluster. **B** Transcription verification of T3SS cluster in the WT, Δ*rpoN* and Δ*rpoN/*pBAD33-*rpoN*^+^ by qRT-PCR. **C** Invasion assay of the WT, Δ*rpoN* and Δ*rpoN*/pBAD33-*rpoN*^+^ strains. Invasion ability of Δ*rpoN* and Δ*rpoN*/pBAD33-*rpoN*.^+^ were reported as percentage relative to the WT. Significant differences were indicated by asterisks (*** *p* ≤ .001; ** *p* ≤ .01; * *p* ≤ .05)

Gram-negative bacteria are known to subvert eukaryotic cell physiological mechanisms using a wide array of virulence factors, among which the T3SS system is often one of the most important. T3SS can remodel cytoskeletal integrity to promote intracellular invasion, as well as silencing specific eukaryotic cell signals, notably to hinder or elude the immune response and cause apoptosis. A previous study reported that RpoN has an important regulatory effect on the T3SS cluster; however, in the transcriptomic profiling of the Δ*rpoN* mutant, we found that the overall change in the expression of the T3SS cluster was not obvious (Fig. 6A), which was verified using qRT-PCR in the WT, Δ*rpoN*, and Δ*rpoN/*pBAD33-*rpoN*^+^ strains (Fig. 6B). The results of the T3SS cluster analysis showed that only a few genes showed differences in expression. However, we still wanted to phenotype the strains by invasion assay to verify whether RpoN affects *P. shigelloides'* ability to infect Caco-2 cells. Invasion assays of the WT, Δ*rpoN*, and Δ*rpoN*/pBAD33-*rpoN*^+^ strains indicated that RpoN had little effect in the ability of *P. shigelloides* to infect Caco-2 cells (Fig. 6C).

## Discussion

*Plesiomonas shigelloides*, which causes intestinal infections, is often isolated from seafood, uncooked food, and contaminated water [59]. To date, few molecular studies on the pathogenic mechanisms of *P. shigelloides* have been published. Transcription factors play a crucial role in microbial growth and the response to environmental changes by regulating the expression of target genes [8]. Sigma factors are the most widely occurring transcription factors, especially, σ^54^ factors acts as multifunctional regulators of many important biological processes. In this study, using RNA sequencing, we found that RpoN regulates approximately 13.2% of the *P. shigelloides* transcriptome, comprising 398 DEGs, which include 210 downregulated genes and 188 upregulated genes. Based on number of the DEGs, the main DEG groups were related to biosynthesis of cofactors, nucleotide metabolism, oxidative phosphorylation, pantothenate and CoA biosynthesis, biosynthesis and metabolism of amino acids, butanoate and glycerophospholipid metabolism, flagellar assembly, bacterial chemotaxis and bacterial secretion system, except for groups with function unknown and general function prediction only. The RNA-seq-dependent transcriptomics analysis indicated that RpoN significantly regulated amino acid biosynthesis and metabolism of nitrogen and carbon in *P. shigelloides*, including valine, leucine, isoleucine, arginine, and lysine biosynthesis, and glycine, serine, threonine, cysteine, and methionine metabolism.

Previous studies have been reported that RpoN controls bacterial growth [60–62], swimming and twitching motility [63]. In this study, the differences in growth between the WT and Δ*rpoN* mutant in LB liquid medium and DMEM were compared, which showed that the growth of the Δ*rpoN* mutant slightly lagged behind that of the WT in the lag phase when grown in LB liquid and lagged behind that of the WT in the log phase when grown in DMEM. The migration in the swimming agar plate of the WT, Δ*rpoN*, and Δ*rpoN/*pBAD33-*rpoN*^+^ strains suggested that RpoN positively regulates the motility of *P. shigelloides*. The TEM results also showed that the absence of RpoN caused failure of flagellar formation in *P. shigelloides*. Furthermore, transcriptome analyses revealed that the expression of more than half of the flagellar genes were reduced in the Δ*rpoN* mutant relative to that in the WT. The results indicated that RpoN is essential for the motility and flagellar synthesis of *P. shigelloides.* However, we also observed that certain flagella genes were upregulated, and some flagella genes were not significantly changed (Fig. S1A and S1B). This cannot change the phenomenon that the Δ*rpoN* mutant was non-motile and lacked flagella. For this, we speculate that the expression of some flagella genes, such as *flaC* and *fliA*_L_ for flagellar synthesis and *motA* for motor assembly, were significantly affected by RpoN and dominantly contributed to the motility of *P. shigelloides.* In subsequent studies we will explore which flagellar genes are directly regulated by RpoN and identify the core regulators of the flagellar hierarchy for *P. shigelloides*.

Bacteria have evolved multiple strategies to survive and develop optimal fitness in their ecological niche. They deploy protein secretion systems for robust and efficient delivery of antibacterial toxins into their target cells, thereby inhibiting their growth or killing them [64]. The T6SS system is a contact-dependent bacterial weapon that allows for direct killing of competitors through the translocation of proteinaceous toxins. Many studies have reported that RpoN also controls the bacterial T6SS [65–68] and virulence [69, 70]. Previous studies have also reported that RpoN directly binds to the promoter regions of *hcpA* and *hcpB*, which encode Hcp1-family T6SS effectors [71]. In the present study, transcriptomic profiling of the Δ*rpoN* mutant revealed a positive regulatory role of RpoN on both the large cluster and auxiliary cluster of the T6SS. In addition, we found RpoN in the DNA pull-down assay previously carried out for the *hcp* gene. Meanwhile, killing assay verified that RpoN contributes to the killing ability of *P. shigelloides* by positively regulating the expression of T6SS clusters. In a subsequent study, we will purify the RpoN protein to further verify which genes are directly regulated by RpoN in the T6SS clusters.

In addition to the two gene clusters of T6SS, we also found another secretion system, T2SS-2, which was positively regulated by RpoN. Two types of T2SS were distributed in *P. shigelloides* genomes, T2SS-1 and T2SS-2. The results of RNA-seq indicated that RpoN positively regulates the T2SS-2 cluster in *P. shigelloides*. However, the expression of T2SS-1 cluster in the transcriptomic profile of Δ*rpoN* mutant was not significantly different. Moreover, the killing assay indicated that deletion of the T2SS-2 cluster reduced the ability of *P. shigelloides* to kill *E. coli* MG1655, suggesting that the T2SS-2 cluster was associated with the killing ability of *P. shigelloides.* Moreover, this is the first report that RpoN positively regulates the expression of T2SS-2. Previous studies reported the RpoN-dependent cascade regulation of T3SS, which proved that RpoN activates hrpL through the interaction between HrpR and HrpS, thereby regulating *hrp* gene transcription [72, 73]. In *Erwinia amylovora* and *Dickeya dadantii*, HrpS, but not HrpR, interacts with RpoN and activates *hrpL*, thus regulating the transcription of T3SS-associated genes [74–76]. However, we found that the overall change in the expression of the T3SS cluster in the transcriptomics profiling of the Δ*rpoN* mutant was not obvious and the invasion assay verified that RpoN had little effect in the ability of *P. shigelloides* to infect Caco-2 cells.

**Fig. 7:**
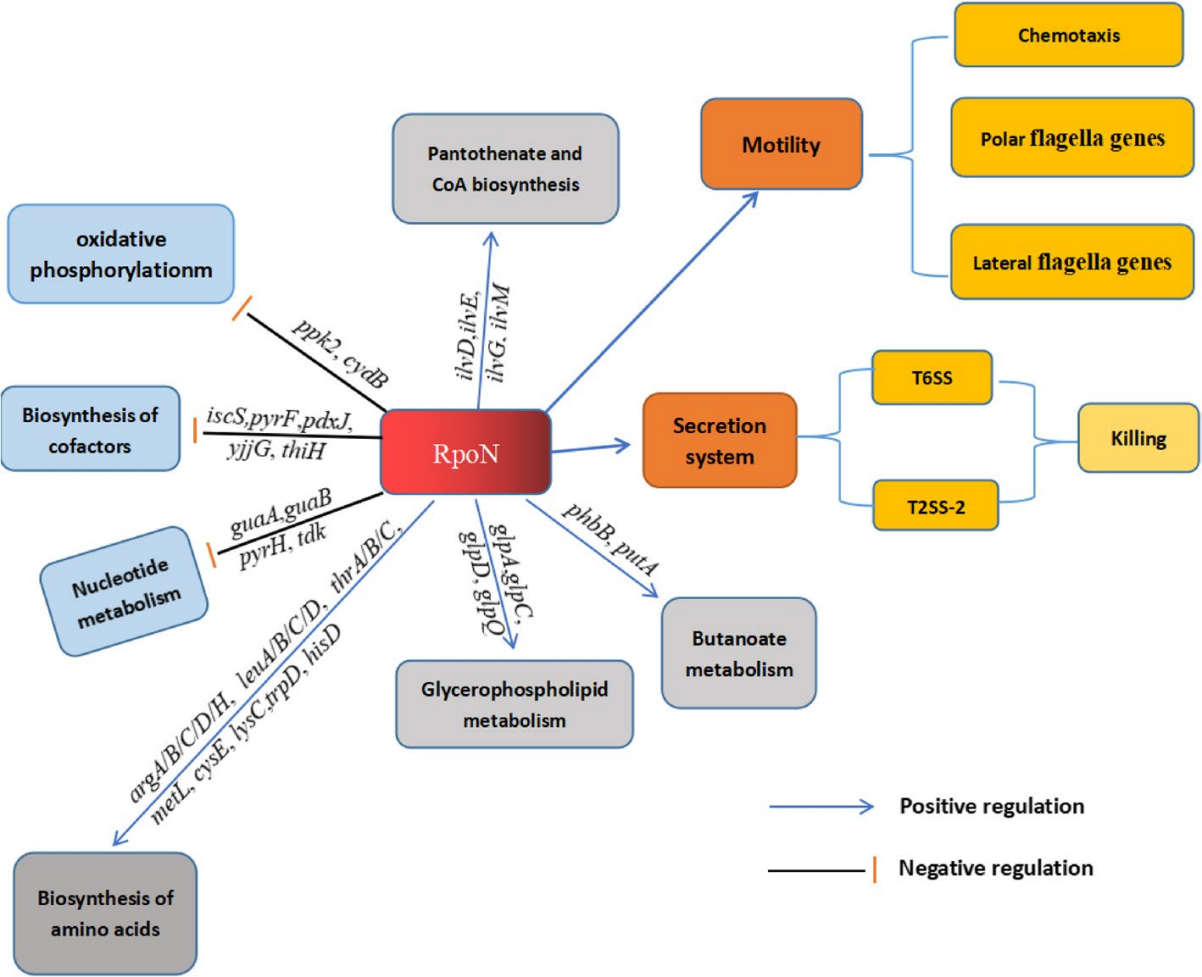
Schematic of the proposed RpoN regulatory mechanism in *P. shigelloides*. The potential regulatory pathways and interplays of RpoN are proposed according to our observations

In the present study, we revealed the RpoN-controlled pathways, and our proposal regarding the major metabolic pathways regulated by RpoN in *P. shigelloides* is outlined in Fig. 7. These findings and knowledge support the key regulatory role of RpoN in bacterial growth and pathogenesis, as well as laying the groundwork for further determination of the complex regulatory network of RpoN in bacteria.

## Conclusions

In this work, the RNA-seq results showed that RpoN regulates ~ 13.2% of the *P. shigelloides* transcriptome, and is involved in amino acid transport and metabolism, glycerophospholipid metabolism, pantothenate and CoA biosynthesis, ribosome biosynthesis, flagellar assembly, and bacterial secretion system. In addition, we showed that T2SS-2 is related to the killing ability of *P. shigelloides*: RpoN is required for the motility and contributes to the killing ability of *P. shigelloides,* and affects the killing ability by positively regulating T6SS and T2SS-2 genes.

## Materials and methods

### Bacterial strains, growth conditions, and plasmids

The bacterial strains, as well as the plasmids used, are listed in Table 1. Bacteria were grown in Dulbecco’s Modified Eagle’s medium (DMEM) supplemented with 20% fetal bovine serum (FBS) and in Luria–Bertani (LB) liquid, solid, and semi-solid medium at 37 ℃ (statically or in a shaking incubator) or at 30 ℃ statically. If necessary, the media were supplemented with ampicillin (25 μg/ml), chloramphenicol (25 μg/ml), or kanamycin (50 μg/ml).

**Table 1:** Bacterial strains and plasmids used in this study

Strains/plasmids	Genotype or relevant characteristics^a^	Source or reference
***Plesiomonas shigelloides* strains**		
G5884	Wild type, serotype O45:H2^b^	CNCTC^b^ Aer 44/89
Δ*rpoN*	*rpoN* gene deletion mutant of G5884	This study
Δ*rpoN*/pBAD33-*rpoN*^+^	Δ*rpoN* containing pBAD33 carrying *rpoN* ORF with its own promoter	This study
WT*/*lux	WT containing pMS402	This study
WT*/flaC*-lux	WT containing pMS402 carrying *flaC* promoter	This study
WT*/motA*-lux	WT containing pMS402 carrying *motA* promoter	This study
WT*/fliA*_L_-lux	WT containing pMS402 carrying *fliA*_L_ promoter	This study
Δ*rpoN/flaC*-lux	Δ*rpoN* containing pMS402 carrying *flaC* promoter	This study
Δ*rpoN/motA*-lux	Δ*rpoN* containing pMS402 carrying *motA* promoter	This study
Δ*rpoN/fliA*_L_-lux	Δ*rpoN* containing pMS402 carrying *fliA*_L_ promoter	This study
ΔT2SS-2	T2SS-2 cluster deletion mutant of G5884	This study
***E. coli* strains**		
DH5α λ*pir*	Transformation host	Lab collection
S17–1 λ*pir*	Tp^R^ Sm^R^ *recA*, *thi*, *pro*, *hsdR*-M^+^RP4: 2-Tc:Mu: Km Tn7 λ*pir*, Km^r^, Sm^r^, Tp^r^	Lab collection
MG1655	F-λ-ilvG- rfb-50 rph-1; Cm^r^	Lab collection
**Plasmids**		
pRE112	Widely used gene knocked vector, with onT RP4; Cm^r^	Lab collection
pBAD33	Arabinose inducible expression vector; Cm^r^	Lab collection
pMS402	For construct promoter-luxCDABE reporter fusion; Km^r^	Lab collection
pRE112-*rpoN*^*−*^	pRE112 containing the homologous arms of *rpoN* gene of G5884; Cm^r^	This study
pRE112*-*T2SS-2^*−*^	pRE112 containing the homologous arms of T2SS-2 cluster of G5884; Cm^r^	This study
pBAD33-*rpoN*^+^	pBAD33 with complete *rpoN*; Cm^r^	This study
pMS402-*flaC*	pMS402 with *flaC* promoter; Km^r^	This study
pMS402-*motA*	pMS402 with *motA* promoter; Km^r^	This study
pMS402-*fliA*_L_	pMS402 with *fliA*_L_ promoter; Km^r^	This study

^a^ r = resistant

^b^ CNCTC, Czech National Collection of Type Cultures, the Czech Republic

### Gene deletion and complementation

In this study, the suicide vector pRE112 was used to make deletion mutations of the *rpoN* gene and the T2SS-2 cluster of *P. shigelloides* [77]. The complementation strain, Δ*rpoN/*pBAD33-*rpoN*^+^, was constructed by introducing the recombinant vector pBAD33-*rpoN*^+^ into the Δ*rpoN* strain via electroporation. Agarose gel electrophoresis and DNA sequencing of PCR products was used to confirm the presence of the correct deletion mutations and complementation strains. Confirmation of the deletion of *rpoN* and T2SS-2 cluster and the complementation of *rpoN* in *P. shigelloides* was shown in Fig. S1D to K. All primers used in this study are shown in Table 2.

**Table 2:** Primers used in this study

Name	Sequence (5′–3′)
**Primers for construction of mutants**	
∆*rpoN*-S-F	^*GCTCTAGA*^CAACCTGATGGCGGTACTG
∆*rpoN*-S-R	CCTGTTGTCT GCAACCTCGGTATCCGTACT
∆*rpoN*-X-F	CCGAGGTTGC AGACAACAGGATGAGGAAGACG
∆*rpoN*-X-R	^*GGGGTACC*^CTGCCCATTTTTTCGCG
∆*rpoN*-F	CAACCTGATGGCGGTACTG
∆*rpoN*-R	CTGCCCATTTTTTCGCG
∆T2SS-2-S-F	*GCTCTAGA*^GTTAGTGAGTCGTCTATGTTGGCTT^
∆T2SS-2-S-R	GCTACGCCATCGTCACCCCG^CCGTTAT^
∆T2SS-2-X-F	CGGGGTGACGATGGCGTAGC^CGGTGGT^
∆T2SS-2-X-R	*GGGGTACC*^CACCACGCCGCCTTGT^
∆T2SS-2-F	GTTAGTGAGTCGTCTATGTTGGCTT
∆T2SS-2-R	CACCACGCCGCCTTGT
**Primers for identification of plasmid**	
pRE112-U-F	CACTGTTCGTCCATTTCCG
pRE112-D-R	TTCGTCTCAGCCAATCCCT
pBAD33-U-F	AACAAAGCGGGACCAAAG
pBAD33-D-R	AGAGCGTTCACCGACAAA
pMS402-U-F	GGTCAAATGAATGCAGGGCT
pMS402-D-R	AGAGTCATTCAATATTGGCAGGT
**Primers for construction of complemented strain**	
pBAD33-*rpoN*^+^-F	GGGGTACCATGAAGCCAAGTTTACAACTCAAG
pBAD33-*rpoN*^+^-R	GCTCTAGATTAAACCAAGCGTTTACGCTG
**Primers for lux**	
lux-*flaC*-F	CCGCTCGAGATTTCTTGACATGCCGCGTT
lux-*flaC*-R	CGGGATCCATCTCCGTTAAACTTGCCGC
lux-*motA*-F	CCGCTCGAGTCCCCAGGTCTCAAAATCGT
lux-motA-R	CGGGATCCCATCAAACCTCTGTGCTCGT
lux-*fliA*_L_-F	CCGCTCGAGGCAAGCTGGCATCTCTGTAC
lux-*fliA*_L_-R	CGGGATCCCTATCCTCTGTCTACCGCGC
Primers for qRT**-**PCR	
*gyrB*-RT-F	GATTTGCGCACTGGGTAGCC
*gyrB*-RT-R	GCGGCTGTTTGGATCCATGG
*flaC*-RT-F	TTGCGATCGACTCATCCCTG
*flaC*-RT-R	CAGGATCTGCTGCTTGGTCA
*motA*-RT-F	GGTGGTTTTTTTCGCAGC
*motA*-RT-R	TTCCTCTTACAAATAAAACGCC
*fliA*_*L*_-RT-F	ATAGGTTTTGCCTTGCGATA
*fliA*_*L*_-RT-R	TCTATCCTCTGTCTACCGCG
*tssB*-RT-F	GTGGTCAGCAGGCCGAAATC
*tssB*-RT-R	TTGGCACCCCTCTTCCAGAC
*tssC*-RT-F	GGGTATTGCTGCGTTGGTGG
*tssC*-RT-R	TGGTGGCATGCAAGACTTGG
*tssE*-RT-F	TGTCATTTCAAGTCACAGCACA
*tssE*-RT-R	GGTCACCTGCTCGATATTCTCCA
*tssF*-RT-F	TTGTACGCGGCGATGAAACG
*tssF*-RT-R	CAAATGACGGTGAGCTGCCC
*tssG*-RT-F	CAATCGCGCCAAGATGCTGT
*tssG*-RT-R	ACCTTATCGCCCAGCACCAA
*tagH*-RT-F	ATCGTGAGCTGGCCAGTCAT
*tagH*-RT-R	GGCTGGGATGCTGTAGGGAT
*tssJ*-RT-F	ATGTGGCGATGAACCCGGAT
*tssJ*-RT-R	CGTAATCGGTGGCCAACAGG
*tssK-*RT-F	TGCTCGCTTAAGTGCCCTGA
*tssK*-RT-R	GACCTCACTGACGCCACTGA
*tssL*-RT-F	ACCATGCCGCACAGCTAGAT
*tssL*-RT-R	TCTGTCACCACGCGCTGATA
*tssH-*RT-F	CGAAAGCAAAGCAAACGCCG
*tssH*-RT-R	CTTCGCCAACCACAATCGGG
*vasH*-RT-F	ATTCACGACCGCCTGACTGT
*vasH*-RT-R	GTGATCCAGTGTGCGCTTCG
*tagO*-RT-F	AGGCTCCTGTGTCGGTTAGC
*tagO*-RT-R	CCAAATGCGGGTAATGGCGT
*tssA*-RT-F	TGCGCGAGATGACCTTCAGT
*tssA*-RT-R	ACTCGCTGCCTAAATCCGCT
*tssM*-RT-F	CAGTTTGAGTGCGGCGGATC
*tssM*-RT-R	TCATTGCCAATCACCGCTGC
*impA*-RT-F	CATTGCAGGGTATGGAGCGC
*impA*-RT-R	CAAAGGCGAGGCTTGCTCAG
*paaR*-RT-F	ATTACGGCCAGCCCTGATGT
*paaR*-RT-R	GCTTATCGTGCGGCTCAAGG
*vgrG1*-RT-F	GGGCATGCGGTGATTGTGTT
*vgrG1*-RT-R	CCTGCCCAGCCTGATCTTCA
*hcp*-RT-F	TCACCTCTGAATCCGTGGGC
*hcp*-RT-R	GCGCAACGGTGAACTGGAAT
*vgrG2*-RT-F	CCGGGTTGTGCGTTCAGTTT
*vgrG2*-RT-R	CACGGTAGCGATTTGTGGGC
orf00078-RT-F	TACGCCTGAGTGATGCCCAG
orf00078-RT-R	TCCTCACTTCCCCACCATGC
orf00080-RT-F	TCGGCCAGGCGATACAACTT
orf00080-RT-R	CGGATCTCACGCCACTCAGT
*yghF*_*2*_-RT-F	CAAGGATAACGTGCTGGTCG
*yghF*_*2*_-RT-R	AATCCGGTAATTCACGCAGC
*gspD*_*2*_-RT-F	CGCCAGATATCATGCAGTCG
*gspD*_*2*_-RT-R	GCACGCCGAAATTGATGTTG
*gspE*_*2*_-RT-F	GCCTTACTCACTAGCGCCTA
*gspE*_*2*_-RT-R	TGGCGTTGATGAGCTTGATG
*gspF*_*2*_-RT-F	TAGCCGAGCAGTGTGAGAAA
*gspF*_*2*_-RT-R	TGGCGCAAAACAACTCATCA
*gspG*_*2*_-RT-F	AGTGATGGTGGTGATCGTGA
*gspG*_*2*_-RT-R	TCGGGTACACGCTGTTATCC
*gspH*_*2*_-RT-F	CATTAGCTGGTCGACAAGCC
*gspH*_*2*_-RT-R	TACTGTCGAGCCACACTTGT
*gspI*_*2*_-RT-F	GTATTGGCGCAGTAAGTCGG
*gspI*_*2*_-RT-R	TCATAGCGCTGACTCCGTAC
*gspJ*_*2*_-RT-F	TCAGGTGGTTGATGGGGTAC
*gspJ*_*2*_-RT-R	ATTGTAAAAGTCCGTCGCCG
*gspK*_*2*_-RT-F	TTAGATGGGCAAGTCACCGT
*gspK*_*2*_-RT-R	CGAACTGATGGCGTTGAACA
*gspL*_*2*_-RT-F	TTTAGACGTGGCCCAGGATT
*gspL*_*2*_-RT-R	ACATCGGGTTTTGTTTCGGG
*gspM*_*2*_-RT-F	TGTTGGTTGGCGGATTGTAC
*gspM*_*2*_-RT-R	CAATTGCCGTAACAGCTGCT
*spaS*-RT-F	CAACCACACCAGTAGACGCA
*spaS*-RT-R	AACGGATGTGAAATTGGCGC
*spaR*-RT-F	TTCGCTGGATCAATCGTGCT
*spaR*-RT-R	GTCATGCTGGGCCTGTTACT
*spaQ*-RT-F	GTTTCACCATACCAGCCCGA
*spaQ*-RT-R	ATGATTTGATGTTTGCGGGCAA
*spaO*-RT-F	GCCAACCACGTGACAACATC
*spaO*-RT-R	GTGACCGTTGATTTGCCACC
*spaN*-RT-F	GCTGCTGATGTGGATTTCGC
*spaN*-RT-R	CAGCCTTCAGATTCGGTGGT
*invC*-RT-F	AGCTATCAAACACCGACGCA
*invC*-RT-R	TTCGCGATCAGGGTAAACGG
*invG*-RT-F	CGCCCATCCAGAGTGCTTAT
*invG*-RT-R	GAGGATCTGGAGCAACTCGG
*invF*-RT-F	ACAACGCGAACATTCACACG
*invF*-RT-R	GCTGGGTGTTGGCAAATGAC
*prgH*-RT-F	TGGCCGACAATTACCCAGAC
*prgH*-RT-R	CGTTTGAGCTCCGTTTCTGC
*prgI*-RT-F	CCGGCGTTGAGGACTTACAA
*prgI*-RT-R	CGGAAGTTTTGTATGATGGCGG
*sipA*-RT-F	TTGACTCGACATTCTGCGCT
sipA-RT-R	CAAATCCGGCCACAAAGCTC
*sipD*-RT-F	GCCTGCCACGCTTGAAATTT
*sipD*-RT-R	TCAGTGATCGGAAGGTTGCC
sipC-RT-F	GCCTCAAGCGCAGACTTACT
*sipC*-RT-R	CTGGGGCAATTGATGGTCCT
*sipB*-RT-F	GCCTCAAGCGCAGACTTACT
*sipB*-RT-R	CTGGGGCAATTGATGGTCCT
*spaT*-RT-F	GCTTCCGCTTCATCGAGTCT
*spaT*-RT-R	GAAATGGTCTGGGAAGCGGT
*spaP*-RT-F	TGCTTTACCACCGGCATCAT
*spaP*-RT-R	GCGTCTGGGACCTGCTTTAT
*invB*-RT-F	CGAGCTCGCCGTCTTGAATA
*invB*-RT-R	AGCATTCACATCAGCCAGCA
*invA*-RT-F	AGTTAACCACGCGCACCATA
*invA*-RT-R	TCAAGGGCCACTGGAAAAGG
*invE*-RT-F	CCAACACCCGCTCAAAACTG
*invE*-RT-R	CAGCGTTTCGTTCAGTCTGC
*invH*-RT-F	ATGCAGCGAGGATGGAGTCA
*invH*-RT-R	ATGCCCGCTCAAATTCGCTA
*hilA*-RT-F	TACAAAATCGCATTGCCGCC
*hilA*-RT-R	AGCTGCAGTACAATGACGCT
*iagB*-RT-F	GCAACGTTATGGTTATAGCTGGG
*iagB*-RT-R	ACGTTCCCAAATTTTGTTAGCA
*prgJ*-RT-F	ATTACACGCCAAGACACCGAT
*prgJ*-RT-R	TCGAGGCGAGCTAAGATTGC
*prgK*-RT-F	AGTCGCCCAGATGTTTCCTG
*prgK*-RT-R	ACGCGGACAAGTGAATGGAT
*orgA*-RT-F	TCAACAACAAGCCGAGCAGA
*orgA*-RT-R	CATGGGCTAGCAACTGTCGA

Underlined letters show Kpn1 、sacI、Xba1、BamhI or XhoI restriction site

F/R: The upstream and downstream primers

S/X-F/R: The upstream and downstream primers for the upstream and downstream gene fragments of *rpoN* or T2SS-2 in the O45 genome

U/D-F/R: Upstream and downstream sequencing primers of plasmid

### Transcriptome sequencing

Cultures of *P. shigelloides* WT and the Δ*rpoN* mutant were grown in LB at 37 ℃ for 12 h to the stationary phase (OD600 ≈ 1.5), and then were harvested using centrifugation. Total RNA of the WT and Δ*rpoN* mutant strains was extracted using the TRIzol® Reagent (Invitrogen) according to the manufacturer's protocol, followed by treatment with an RNase-Free DNase. RNA degradation and contamination was monitored using 1% agarose gels. The total amount and integrity of the RNA were assessed using an RNA Nano 6000 Assay Kit on the Bioanalyzer 2100 system. cDNA was prepared and modified according to the manufacturer’s protocol. After the cDNA library was tested and qualified, it was sequenced on an Illumina NovaSeq 6000 platform (Illumina, San Diego, CA, USA) to generate 150 bp paired-ends reads. Three independent libraries were prepared for each of the RNA-seq samples. After filtering the raw reads, the clean reads were mapped to the genome of *P. shigelloides* (GenBank assembly accession GCA_009183595.1). HTSeq (v0.9.1) was used to quantify of gene expression levels, and then the Fragments Per Kilobase of transcript sequence per Millions base pairs sequenced (FPKM) value of each gene was calculated based on the length of the gene and read count mapped to this gene. The criteria for a significant difference in expression were |log2 fold change|≥ 1 and adjusted P-value (padj) ≤ 0.05. Gene ontology (GO) and Kyoto Encyclopedia of Genes and Genomes (KEGG) pathway databases [78–80] were used to analyze the functions and enriched pathways of the DEGs.

### Quantitative real-time reverse transcription PCR (qRT-PCR)

To confirm the RNA-Seq results, differentially expressed genes (DEGs) from the RNA-Seq analysis related to the observed phenotypic changes were selected, and qRT-PCR was performed to verify their expression changes. Briefly, Total RNA of the WT, Δ*rpoN* and Δ*rpoN/*pBAD33-*rpoN*^+^ strains was extracted using the TRIzol® Reagent (Invitrogen), followed by dissolution in RNase-Free water. cDNA synthesis was performed by using a PrimeScript™ RT reagent Kit (Takara) with 1.2 μg total RNAs in each reaction mix. Gene specific primers for the qRT-PCR are listed in Table 2. Using the cDNA as the template, qPCR analysis was conducted on an Applied Biosystems ABI 7500 sequence detection system (Applied Biosystems, Foster City, CA, USA) with the SYBR green fluorescence dye. The *P. shigelloides* *gyrB* gene was used as the internal control for qRT-PCR, and the relative expression levels were calculated as fold change values using the 2^−△△CT^ method [81]. Each experiment was carried out in triplicate.

### Motility assay and transmission electron microscopy of flagella

To measure motility, motility assays of *P. shigelloides* WT, Δ*rpoN*, and Δ*rpoN*/pBAD33-*rpoN*^+^ strains were performed as described previously [82]. Briefly, freshly grown bacterial colonies were transferred using a sterile toothpick onto swimming agar plates. The swimming agar plates were incubated for 12 h at 30 °C and motility was examined by the migration of bacteria through the agar from the center toward the plate periphery. We conducted the experiments at three time points with six repetitions for each time. Transmission electron microscopy (TEM) and negative staining were used to visualize the flagella of the WT, Δ*rpoN*, and Δ*rpoN*/pBAD33-*rpoN*^+^ strains, as previously described [83].

### Luminescence screening assay

The procedures of the lux reporter assay were described in a previous study [84]. The amplification products of the respective promoter regions were digested and cloned into the XhoI-BamHI site, upstream of the lux genes, in the plasmid pMS402. Briefly, *flaC*, *motA*, and *fliA*_*L*_ promoter-lux fusions were constructed in the Δ*rpoN* mutant and WT, and bacterial cultures were grown in LB medium at 37 °C to the mid-logarithmic phase. The cultures were transferred into a black 96-well plate with a transparent bottom. Promoter activities was measured at OD600 using a Synergy 2 plate reader (Agilent BioTek, Santa Clara, CA, USA). Primers for the lux reporter assay are listed in Table 2. Each experiment was carried out in triplicate.

### Growth assay

Growth assays were performed as described previously [85]. The WT, Δ*rpoN*, and Δ*rpoN*/pBAD33-*rpoN*^+^ strains were cultured overnight at 37 °C with shaking in sterile LB medium. Then, the bacterial solution was added to five wells of a 96-well cell plate containing 200 μl of LB or DMEM at a ratio of 1:200 per well. Fresh LB or DMEM was added to the surrounding wells as controls. Finally, the prepared 96-well cell plate was placed in a Molecular Devices Spectramax 190 full-wavelength microplate reader (Molecular Devices LLC, San Jose, CA, USA) to carry out the dynamic growth experiment. We conducted the experiments at three time points with five repetitions for each time.

### Killing assay

The *E. coli* MG1655 killing assay was carried out as described previously [86], with some modifications. Overnight cultures were diluted (1:100) in LB medium and grown aerobically at 37 °C until the optical density at 600 nm reached 1.5 for *P. shigelloides* and *E. coli*, respectively. The cells were harvested and concentrated. The predator and prey bacteria were mixed at a ratio of 1:1, and 20 µl of this mixture was placed onto LB agar plates without antibiotics. After 3 h of static incubation at 37 °C, the bacteria were removed from the LB agar plates by vortexing in 3 ml of phosphate buffered saline (PBS), and serial dilutions were spotted onto antibiotic-containing LB agar plates to enumerate colony forming units. The killing ability of Δ*rpoN*, Δ*rpoN*/pBAD33-*rpoN*^+^, and ΔT2SS-2 strains were reported as a percentage relative to that of the WT. The experiments were performed at least three times.

### Invasion assay

The invasion assay was carried out as described previously [87]. Briefly, the WT, Δ*rpoN*, and Δ*rpoN*/pBAD33-*rpoN*^+^ strains were grown overnight in LB. The next day, the overnight bacterial solution was transferred to fresh LB and the bacteria were grown to OD_600_ = 0.6. Approximately 5 × 10^7^ the WT, Δ*rpoN*, and Δ*rpoN/*pBAD33-*rpoN*^+^ bacterial cells were layered onto confluent monolayers of approximately 1 × 10^5^ Caco-2 cells (a human epithelial cell line originally derived from colon carcinoma) per well in 24-well plates. The plates were centrifuged at 1000 × *g* for 30 s to promote sinking of the bacteria, followed by incubation at 37 °C in 5% CO_2_ for 1 h. The monolayer was washed extensively with PBS, and fresh, pre-warmed DMEM containing gentamycin was added to kill extracellular bacteria. After 1 h of incubation, the monolayer was washed with PBS twice, and the cells were lysed using 0.1% Triton X-100 for 10 min. The released intracellular bacteria were enumerated using the plate counting method. The invasive ability was expressed as the percentage of the inoculum that survived the gentamycin treatment. We conducted the assay at four time points with six repetitions for each time.

### Statistical analysis

Statistical analysis of the data was performed using GraphPad Prism v7.0 software (GraphPad Inc., La Jolla, CAS, USA) [88]. All data are expressed as means ± standard deviation (SD). Differences between two groups were calculated using independent-samples *t*-test or Mann–Whitney *U* test. A probability value (*P*) ≤ 0.05 was considered statistically significant (in the figures, *** *p* ≤ 0.001; ** *p* ≤ 0.01; * *p* ≤ 0.05; ns indicates not significant). Construction of the RpoN evolutionary tree used the Molecular Evolutionary Genetics Analysis (MEGA 6.0) software package [89].

## Supplementary Information

**Additional file 1.**

## Data Availability

The datasets generated and/or analysed during the current study are available in the Sequence Read Archive, with accession numbers PRJNA902854.

## Abbreviations

RNA-Seq: RNA transcriptome sequencing
DEGs: Differential expressed genes
GO: GeneOntology
KEGG: Kyoto Encyclopedia of Genes and Genomes
WT: Wild-type; Δ*rpoN*, *rpoN* isogenic deletion mutant strain
Δ*rpoN*/pBAD33-*rpoN*^+^: Complementation strain of *rpoN*
LB: Luria–Bertani
DMEM: Dulbecco’s Modified Eagle’s medium
PBS: Phosphate-buffered saline
T2SS: Type II secretion system
T6SS: Type VI secretion system
qRT-PCR: Quantitative real time Polymerase Chain Reaction
