# Supplementary material for: RpoN is required for the motility and contributes to the killing ability of Plesiomonas shigelloides
Source: BMC Microbiol. 2022 Dec 12;22:299. doi: 10.1186/s12866-022-02722-8 (PMC9743648; doi:10.1186/s12866-022-02722-8)
Supplement: Supplementary file 1 — Additional file 1. [file 12866_2022_2722_MOESM1_ESM.docx]

**
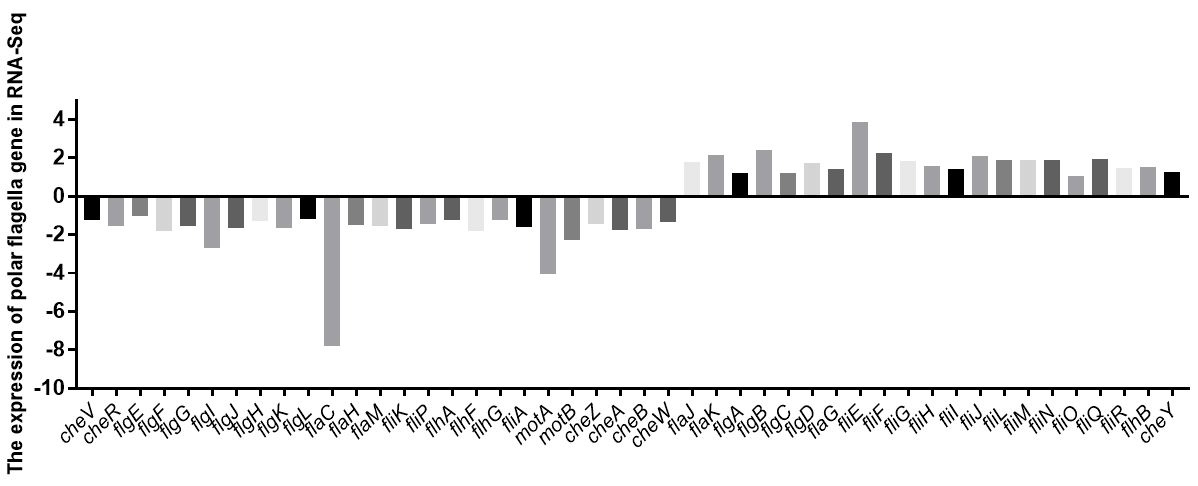
Fig. S1A**

Fig. S1A RNA-seq analysis of the transcription levels of polar flagella genes.

**Fig. S1B**


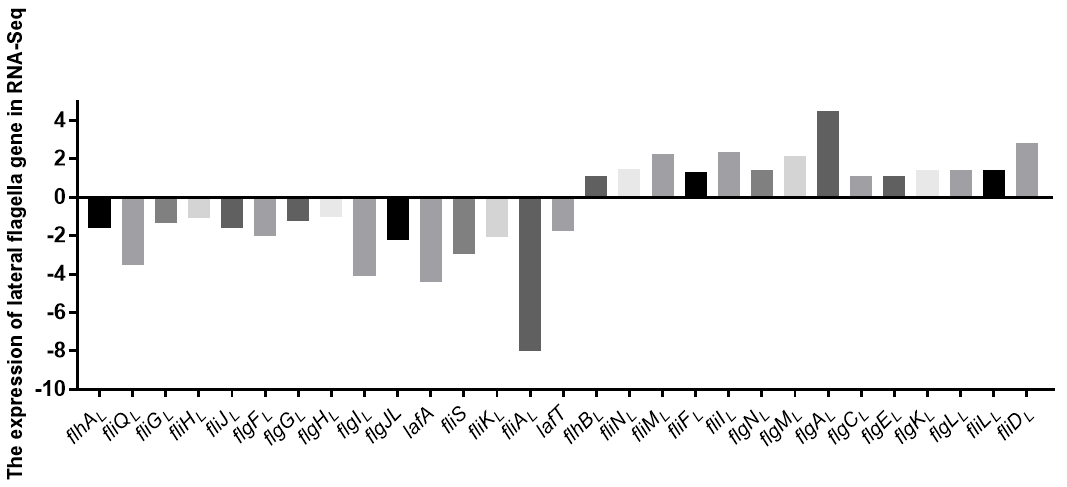


Fig. S1B RNA-seq analysis of the transcription levels of lateral flagella genes.

**Fig. S1C**


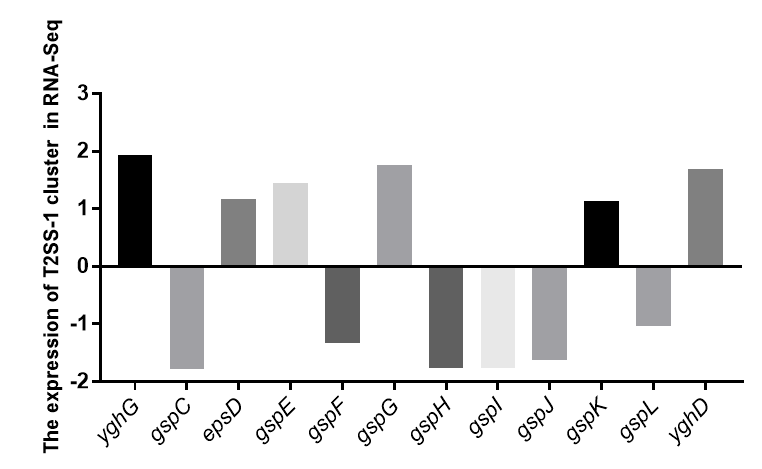


Fig. S1C RNA-seq analysis of the transcription levels of T2SS-1 cluster.

**Fig. S1D**


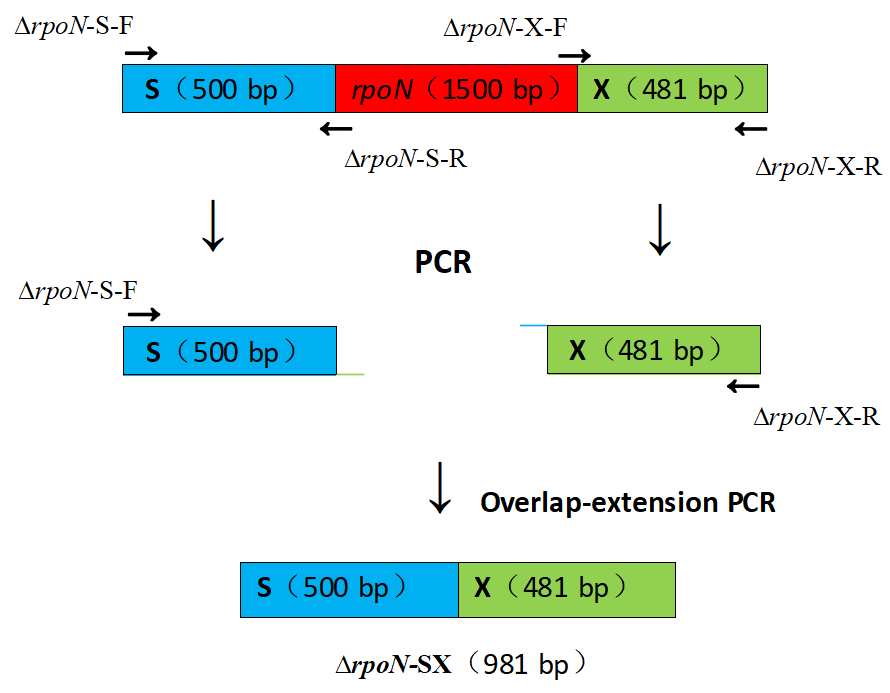


Fig. S1D Graphical process of the deletion of the *rpoN* gene.

**Fig. S1E**


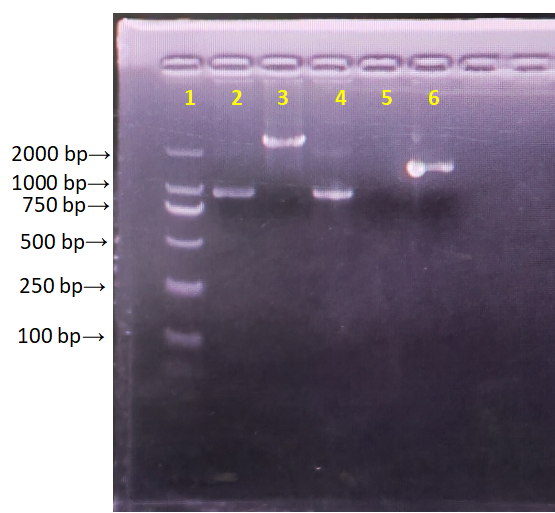


Fig. S1E Confirmation of the deletion of *rpoN* in *P. shigelloides*. 1, DL2000 DNA marker; 2, PCR fragment of ∆*rpoN*-SX (981 bp); 3, PCR amplicon of S‑*rpoN*-X (2481 bp) from the WT genomic DNA; 4, PCR amplificon of ∆*rpoN*-SX from the Δ*rpoN* genome DNA; 5, PCR amplification of *rpoN* from the Δ*rpoN* genome DNA; 6, PCR amplification of *rpoN* (1500 bp) from the WT genome DNA.

**Fig. S1F**


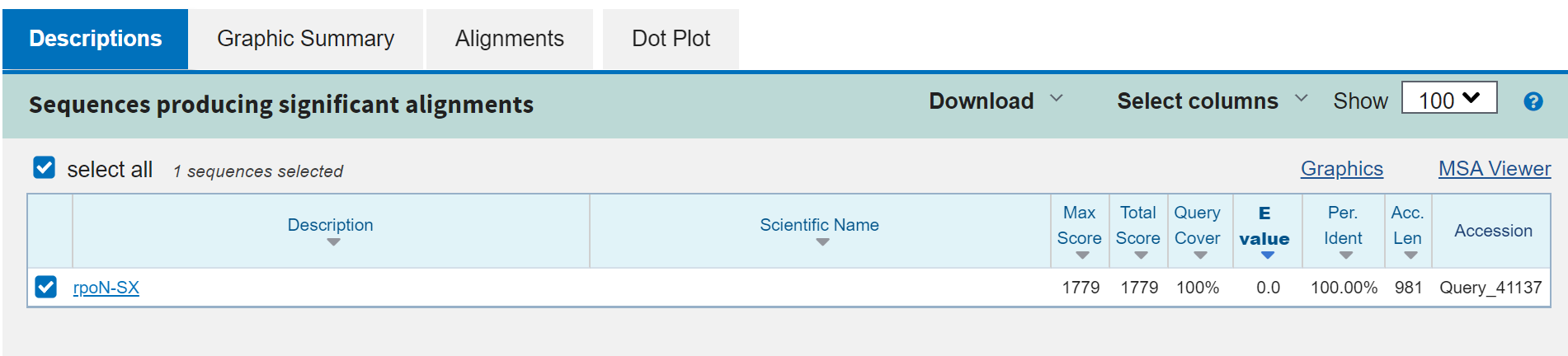


Fig. S1F DNA sequencing of ∆*rpoN*-SX from the Δ*rpoN* genome DNA was performed and aligned to the PCR fragment of ∆*rpoN*-SX (981 bp).

**Fig. S1G**


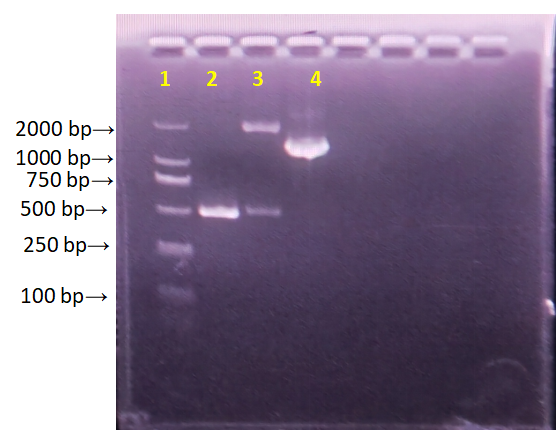


Fig. S1G Confirmation of the omplementation of *rpoN* in *P. shigelloides.*1, DL2000 DNA marker; 2, PCR amplification of pBAD33-UD (500 bp) from the pBAD33 plasmid; 3, PCR amplification of pBAD33-U-*rpoN*-D (2000 bp) from the Δ*rpoN*/pBAD33-*rpoN*^+^ complementation strain; 4, PCR amplification of *rpoN* (1500 bp) from the complementation strain.

**Fig. S1H**


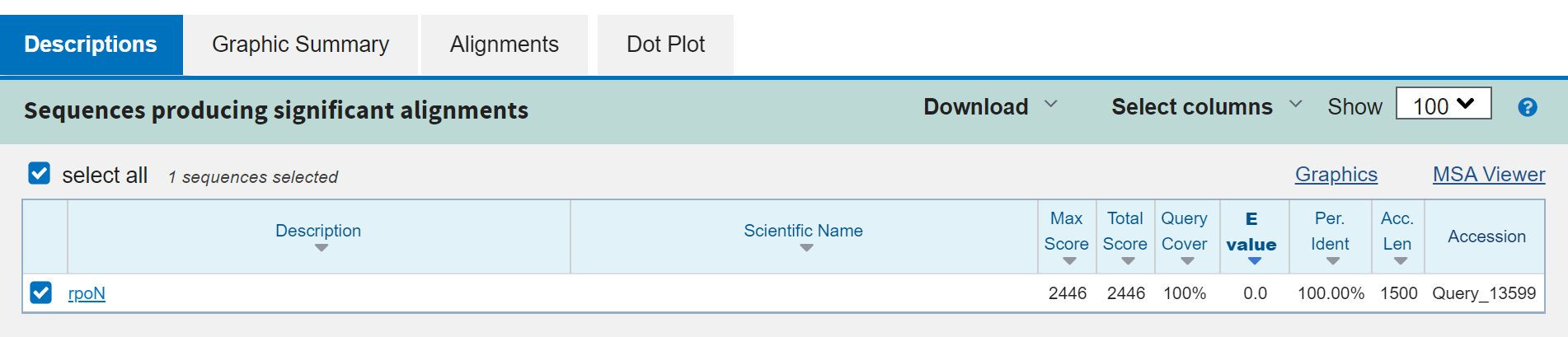


Fig. S1H DNA sequencing of *rpoN* from the Δ*rpoN*/pBAD33-*rpoN*^+^ complementation strain was performed and aligned to the PCR fragment of *rpoN* from the WT genome DNA (1500 bp).

**Fig. S1I**


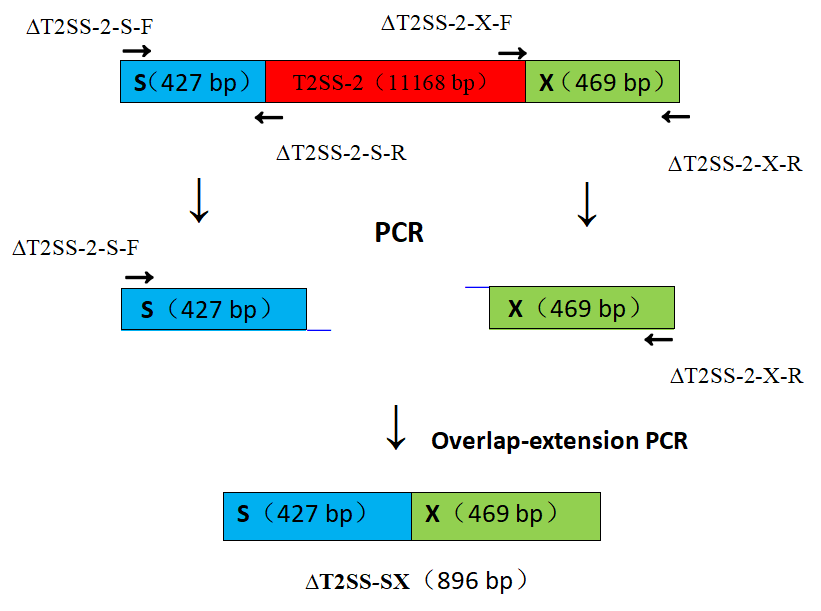


Fig. S1I Graphical process of the deletion of the T2SS-2 cluster.

**Fig. S1J**


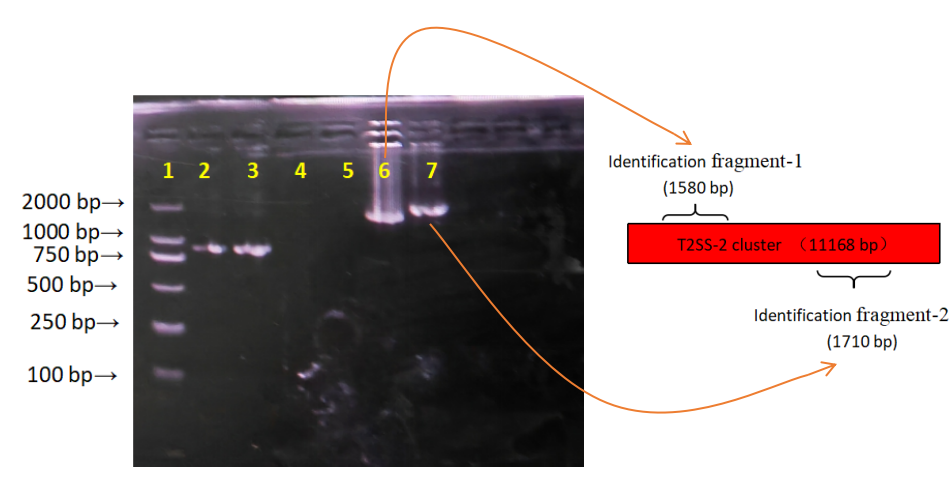


Fig. S1J Confirmation of the deletion of T2SS-2 cluster in *P. shigelloides.* 1, DL2000 DNA marker; 2, PCR of ∆T2SS-2-SX (896 bp); 3, PCR amplificon of ∆T2SS-2-SX from the ΔT2SS-2 genome DNA; 4, PCR amplificon of identification fragment-1 from the ΔT2SS-2 genome DNA; 5, PCR amplificon of identification fragment-2 from the ΔT2SS-2 genome DNA; 6, PCR amplificon of identification fragment-1 (1580 bp) from the WT genome DNA; 7, PCR amplificon of identification fragment-2 (1710 bp) from the WT genome DNA.

**Fig. S1K**


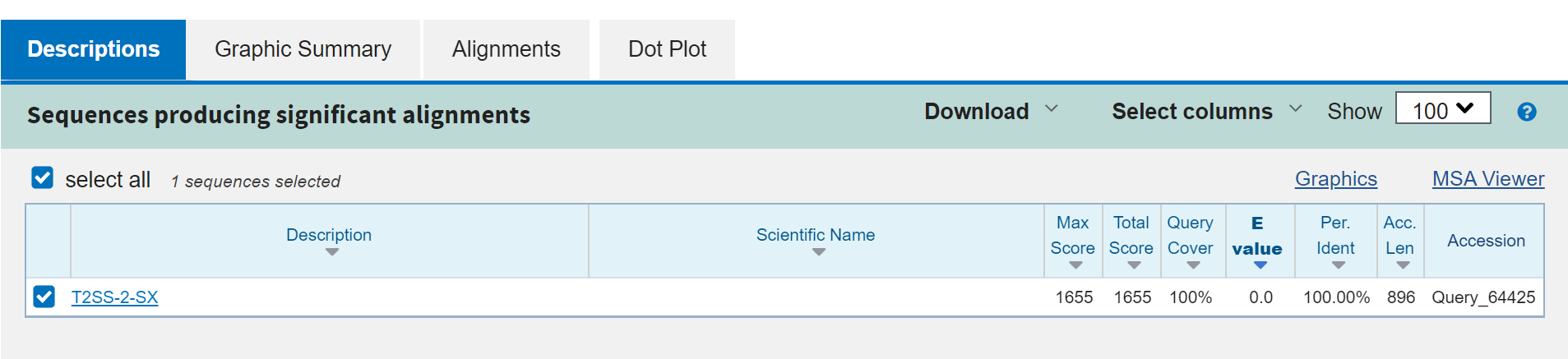


Fig. S1K DNA sequencing of ∆T2SS-2-SX from the ΔT2SS-2 genome DNA was performed and aligned to the PCR fragment of ∆T2SS-2-SX (896 bp).

**Table S1A** Transcriptome revealed differential expressed genes related to RpoN of the *P. shigelloides*

| Gene id | Gene name | log2FoldChange | pvalue | padj |
| --- | --- | --- | --- | --- |
| GBN32_RS12105 | *glpA* | -5.074295353 | 1.64E-16 | 2.48E-13 |
| GBN32_RS12115 | *glpB* | -4.962900644 | 4.87E-15 | 4.91E-12 |
| GBN32_RS15535 | GBN32_RS15535 | -3.562251769 | 5.91E-14 | 4.47E-11 |
| GBN32_RS01165 | GBN32_RS01165 | 3.349897539 | 1.49E-13 | 9.04E-11 |
| GBN32_RS12385 | *zupT* | -3.955938229 | 1.98E-13 | 9.96E-11 |
| GBN32_RS12120 | *glpC* | -3.587221786 | 4.35E-13 | 1.88E-10 |
| GBN32_RS12100 | *glpT* | -3.609736031 | 2.86E-12 | 1.08E-09 |
| GBN32_RS12425 | GBN32_RS12425 | -2.99938867 | 4.68E-11 | 1.57E-08 |
| GBN32_RS03515 | GBN32_RS03515 | -2.95970802 | 1.34E-10 | 4.04E-08 |
| GBN32_RS04310 | GBN32_RS04310 | -3.241939751 | 2.61E-09 | 6.88E-07 |
| GBN32_RS15360 | *pspA* | -2.702910179 | 2.73E-09 | 6.88E-07 |
| GBN32_RS12415 | *ilvA* | -2.602473907 | 5.25E-09 | 1.22E-06 |
| GBN32_RS10875 | *ydfZ* | 2.502488177 | 8.66E-09 | 1.87E-06 |
| GBN32_RS10990 | *rpsJ* | -2.451883061 | 3.67E-08 | 7.40E-06 |
| GBN32_RS11000 | *rplD* | -2.395635184 | 4.05E-08 | 7.67E-06 |
| GBN32_RS04330 | GBN32_RS04330 | 2.754929532 | 1.63E-07 | 2.90E-05 |
| GBN32_RS04320 | GBN32_RS04320 | -2.510493666 | 2.00E-07 | 3.35E-05 |
| GBN32_RS12430 | *ilvM* | -2.675829248 | 2.36E-07 | 3.76E-05 |
| GBN32_RS11005 | *rplW* | -2.284062873 | 2.49E-07 | 3.76E-05 |
| GBN32_RS12420 | *ilvD* | -2.25268777 | 3.06E-07 | 4.41E-05 |
| GBN32_RS10500 | *tagO* | -2.268933099 | 3.29E-07 | 4.44E-05 |
| GBN32_RS05190 | *gspL* | -2.445941897 | 3.38E-07 | 4.44E-05 |
| GBN32_RS11010 | *rplB* | -2.142482206 | 6.14E-07 | 7.60E-05 |
| GBN32_RS12435 | *ilvG* | -2.207892951 | 6.49E-07 | 7.60E-05 |
| GBN32_RS11020 | *rplV* | -2.162964216 | 6.53E-07 | 7.60E-05 |
| GBN32_RS11015 | *rpsS* | -2.165074533 | 6.84E-07 | 7.66E-05 |
| GBN32_RS05045 | *glpD* | -2.202831805 | 7.09E-07 | 7.67E-05 |
| GBN32_RS14875 | *thrC* | -2.134540495 | 1.64E-06 | 0.000165593 |
| GBN32_RS14740 | *leuB* | -2.143954351 | 1.64E-06 | 0.000165593 |
| GBN32_RS10505 | *tssA* | -2.117559803 | 1.73E-06 | 0.000168729 |
| GBN32_RS10995 | *rplC* | -2.068474655 | 1.87E-06 | 0.000176907 |
| GBN32_RS17260 | *fabA* | 2.0346377 | 2.25E-06 | 0.000205923 |
| GBN32_RS15130 | GBN32_RS15130 | 2.020842701 | 2.34E-06 | 0.00020828 |
| GBN32_RS05155 | *gspE* | -2.116950224 | 2.67E-06 | 0.000224312 |
| GBN32_RS13800 | GBN32_RS13800 | 2.005807538 | 2.68E-06 | 0.000224312 |
| GBN32_RS15940 | *iscR* | 2.01309191 | 2.74E-06 | 0.000224312 |
| GBN32_RS11025 | *rpsC* | -1.997591975 | 3.15E-06 | 0.00025095 |
| GBN32_RS11255 | *argB* | -2.031185638 | 3.30E-06 | 0.000256265 |
| GBN32_RS14735 | *leuC* | -1.98019951 | 4.60E-06 | 0.000348146 |
| GBN32_RS15945 | GBN32_RS15945 | 1.941214199 | 5.33E-06 | 0.000393702 |
| GBN32_RS10485 | *icmH* | -2.126091872 | 7.56E-06 | 0.000544395 |
| GBN32_RS10510 | *tssM* | -1.909569482 | 8.34E-06 | 0.00058707 |
| GBN32_RS14810 | GBN32_RS14810 | -1.875110201 | 1.04E-05 | 0.00071625 |
| GBN32_RS10490 | *tssH* | -1.884905204 | 1.07E-05 | 0.00071625 |
| GBN32_RS14350 | GBN32_RS14350 | -2.837765783 | 1.15E-05 | 0.00075963 |
| GBN32_RS11030 | *rplP* | -1.870229469 | 1.23E-05 | 0.000774271 |
| GBN32_RS10515 | GBN32_RS10515 | -1.897802099 | 1.24E-05 | 0.000774271 |
| GBN32_RS10480 | *tssK* | -1.896514902 | 1.25E-05 | 0.000774271 |
| GBN32_RS11280 | *metF* | -2.735134595 | 1.37E-05 | 0.000831317 |
| GBN32_RS14720 | GBN32_RS14720 | 1.888199369 | 1.43E-05 | 0.000848707 |
| GBN32_RS12735 | *flgA* | 2.172842453 | 1.64E-05 | 0.000956295 |
| GBN32_RS14840 | GBN32_RS14840 | 1.943295865 | 1.78E-05 | 0.001015016 |
| GBN32_RS08540 | GBN32_RS08540 | 1.960874429 | 1.86E-05 | 0.001042567 |
| GBN32_RS06260 | *cadB* | -1.843857789 | 2.01E-05 | 0.001106631 |
| GBN32_RS10470 | *tagH* | -1.821952648 | 2.29E-05 | 0.001234811 |
| GBN32_RS03550 | *fliE* | 1.946971327 | 2.35E-05 | 0.001240152 |
| GBN32_RS05175 | *gspI* | -3.125697698 | 2.38E-05 | 0.001240152 |
| GBN32_RS11650 | GBN32_RS11650 | -1.938011086 | 2.87E-05 | 0.001463838 |
| GBN32_RS10495 | GBN32_RS10495 | -1.81951728 | 2.90E-05 | 0.001463838 |
| GBN32_RS12820 | GBN32_RS12820 | 1.773860692 | 3.18E-05 | 0.001577618 |
| GBN32_RS05180 | *gspJ* | -2.563664726 | 3.34E-05 | 0.00160791 |
| GBN32_RS10440 | GBN32_RS10440 | -1.759010445 | 3.35E-05 | 0.00160791 |
| GBN32_RS12065 | *asd* | -1.755585558 | 3.86E-05 | 0.001804091 |
| GBN32_RS05160 | *gspF* | -2.036937453 | 3.88E-05 | 0.001804091 |
| GBN32_RS10520 | GBN32_RS10520 | -1.843716528 | 4.75E-05 | 0.002175703 |
| GBN32_RS08035 | GBN32_RS08035 | -1.836420482 | 5.50E-05 | 0.00248448 |
| GBN32_RS12095 | *glpQ* | -1.86658134 | 6.86E-05 | 0.003050906 |
| GBN32_RS10525 | GBN32_RS10525 | -1.701884043 | 8.31E-05 | 0.003645337 |
| GBN32_RS05870 | GBN32_RS05870 | 1.805470238 | 8.54E-05 | 0.00369188 |
| GBN32_RS11035 | *rpmC* | -1.688928714 | 9.32E-05 | 0.003971614 |
| GBN32_RS03940 | GBN32_RS03940 | 1.868693954 | 0.000110795 | 0.004647035 |
| GBN32_RS17135 | GBN32_RS17135 | 2.41406777 | 0.000112106 | 0.004647035 |
| GBN32_RS13365 | GBN32_RS13365 | -1.971214965 | 0.000116326 | 0.00471593 |
| GBN32_RS12670 | GBN32_RS12670 | -2.130716035 | 0.000116885 | 0.00471593 |
| GBN32_RS08385 | *lnt* | -1.751697507 | 0.000118796 | 0.004729971 |
| GBN32_RS16050 | *guaA* | 1.614159516 | 0.000134379 | 0.005280933 |
| GBN32_RS08735 | GBN32_RS08735 | 1.632563438 | 0.000143606 | 0.005571196 |
| GBN32_RS05185 | *gspK* | -1.847709336 | 0.000152826 | 0.005853823 |
| GBN32_RS04060 | GBN32_RS04060 | 1.968349148 | 0.000168126 | 0.006359356 |
| GBN32_RS06010 | *trmB* | 1.841083664 | 0.000176833 | 0.006606128 |
| GBN32_RS05900 | *speF* | 1.578154279 | 0.000187575 | 0.00692196 |
| GBN32_RS05345 | GBN32_RS05345 | -1.6163913 | 0.000190946 | 0.006939454 |
| GBN32_RS11680 | *recG* | -1.839485536 | 0.000192635 | 0.006939454 |
| GBN32_RS14815 | *gltB* | -1.547525615 | 0.00023194 | 0.008257048 |
| GBN32_RS02220 | *rpoC* | -1.542127769 | 0.000238852 | 0.008404263 |
| GBN32_RS08730 | GBN32_RS08730 | 2.015235672 | 0.000280917 | 0.009680176 |
| GBN32_RS06960 | *aceF* | -1.55680853 | 0.000284495 | 0.009680176 |
| GBN32_RS09745 | GBN32_RS09745 | 2.111508247 | 0.000284711 | 0.009680176 |
| GBN32_RS04000 | GBN32_RS04000 | 1.609551315 | 0.000293865 | 0.009880393 |
| GBN32_RS06015 | GBN32_RS06015 | 1.533908301 | 0.000327454 | 0.010888747 |
| GBN32_RS11310 | GBN32_RS11310 | -2.104376824 | 0.000336548 | 0.011069518 |
| GBN32_RS05015 | *livG* | -1.919277689 | 0.000347222 | 0.011297767 |
| GBN32_RS15480 | GBN32_RS15480 | 1.529398352 | 0.00038455 | 0.012379231 |
| GBN32_RS03365 | GBN32_RS03365 | -1.857210955 | 0.000425695 | 0.013559491 |
| GBN32_RS00445 | GBN32_RS00445 | -1.493119913 | 0.00045211 | 0.014250878 |
| GBN32_RS14730 | *leuD* | -1.506551034 | 0.000475944 | 0.014709838 |
| GBN32_RS04140 | GBN32_RS04140 | -2.077370701 | 0.000480429 | 0.014709838 |
| GBN32_RS11430 | *cysE* | -1.614774676 | 0.000481254 | 0.014709838 |
| GBN32_RS17925 | GBN32_RS17925 | 2.462583983 | 0.000495363 | 0.014989697 |
| GBN32_RS11410 | GBN32_RS11410 | 1.490341542 | 0.000503203 | 0.01507617 |
| GBN32_RS09620 | *pyrF* | 1.818311206 | 0.000511591 | 0.015177214 |
| GBN32_RS17080 | GBN32_RS17080 | 1.511482953 | 0.000568032 | 0.01668801 |
| GBN32_RS12175 | GBN32_RS12175 | -1.69231192 | 0.000583639 | 0.016981652 |
| GBN32_RS00765 | GBN32_RS00765 | 2.153066692 | 0.000634842 | 0.018241424 |
| GBN32_RS09655 | GBN32_RS09655 | 1.610035294 | 0.000638992 | 0.018241424 |
| GBN32_RS05330 | GBN32_RS05330 | -1.44359485 | 0.000661558 | 0.0187091 |
| GBN32_RS05455 | GBN32_RS05455 | -1.421195797 | 0.000698611 | 0.019574052 |
| GBN32_RS08065 | *raiA* | 1.415017151 | 0.000720836 | 0.020011478 |
| GBN32_RS18000 | GBN32_RS18000 | 1.489455354 | 0.00073534 | 0.020228534 |
| GBN32_RS04275 | *lolB* | 1.631478305 | 0.000805538 | 0.021932766 |
| GBN32_RS07735 | GBN32_RS07735 | 1.475179299 | 0.000811788 | 0.021932766 |
| GBN32_RS04890 | *ruvC* | -1.422795854 | 0.000823212 | 0.022044593 |
| GBN32_RS01465 | *rplT* | -1.400337761 | 0.000833128 | 0.022076325 |
| GBN32_RS14870 | *thrB* | -1.515621686 | 0.000844364 | 0.022076325 |
| GBN32_RS01150 | GBN32_RS01150 | 1.610447926 | 0.000846283 | 0.022076325 |
| GBN32_RS08810 | *artQ* | -1.395749722 | 0.000879049 | 0.022735051 |
| GBN32_RS08560 | *phbB* | -1.376483548 | 0.00104449 | 0.026784964 |
| GBN32_RS11700 | *fabY* | -1.503601147 | 0.001087245 | 0.027647085 |
| GBN32_RS11900 | GBN32_RS11900 | -1.439400038 | 0.001129045 | 0.028470756 |
| GBN32_RS04960 | GBN32_RS04960 | -1.36857591 | 0.001173664 | 0.029351304 |
| GBN32_RS12815 | *glmS* | 1.360682315 | 0.001205565 | 0.029901964 |
| GBN32_RS16260 | GBN32_RS16260 | 1.422444199 | 0.001226365 | 0.030170585 |
| GBN32_RS15930 | *suhB* | -1.455046125 | 0.001297139 | 0.031654377 |
| GBN32_RS10465 | *tssG* | -1.365723524 | 0.001344409 | 0.032545452 |
| GBN32_RS13950 | GBN32_RS13950 | 3.905488797 | 0.001369576 | 0.032891552 |
| GBN32_RS11420 | *cpxA* | -1.591216819 | 0.001390143 | 0.033122627 |
| GBN32_RS06265 | GBN32_RS06265 | -1.333787088 | 0.001434427 | 0.033910762 |
| GBN32_RS10700 | GBN32_RS10700 | -1.849239588 | 0.0014637 | 0.034334545 |
| GBN32_RS02925 | GBN32_RS02925 | 1.364102022 | 0.001531043 | 0.035637962 |
| GBN32_RS14890 | *grcA* | 1.321443529 | 0.001566664 | 0.03618874 |
| GBN32_RS10460 | *tssF* | -1.344206227 | 0.001594361 | 0.036324871 |
| GBN32_RS02285 | *lysC* | -1.417443927 | 0.001608396 | 0.036324871 |
| GBN32_RS10475 | *tssJ* | -1.476599693 | 0.001620494 | 0.036324871 |
| GBN32_RS09895 | GBN32_RS09895 | 1.482044568 | 0.001620574 | 0.036324871 |
| GBN32_RS06955 | *aceE* | -1.33255009 | 0.001668602 | 0.036591082 |
| GBN32_RS05165 | *gspG* | -1.499973051 | 0.001672873 | 0.036591082 |
| GBN32_RS01250 | GBN32_RS01250 | 1.338714469 | 0.001681195 | 0.036591082 |
| GBN32_RS04765 | GBN32_RS04765 | -1.382216939 | 0.001690535 | 0.036591082 |
| GBN32_RS01100 | GBN32_RS01100 | -1.408695016 | 0.001692912 | 0.036591082 |
| GBN32_RS07065 | GBN32_RS07065 | 1.379058736 | 0.00173318 | 0.037195762 |
| GBN32_RS08220 | *bolA* | 1.36934685 | 0.001870735 | 0.039865099 |
| GBN32_RS05195 | GBN32_RS05195 | -1.69558945 | 0.001934001 | 0.040788358 |
| GBN32_RS08790 | GBN32_RS08790 | -1.351334266 | 0.00194295 | 0.040788358 |
| GBN32_RS08380 | GBN32_RS08380 | 1.292003721 | 0.001958199 | 0.040788358 |
| GBN32_RS01255 | GBN32_RS01255 | 1.303364471 | 0.001967978 | 0.040788358 |
| GBN32_RS16540 | *sodB* | 1.290343982 | 0.002030579 | 0.04179954 |
| GBN32_RS07225 | GBN32_RS07225 | -1.670009564 | 0.002067529 | 0.04210442 |
| GBN32_RS00145 | GBN32_RS00145 | 1.63097161 | 0.002073218 | 0.04210442 |
| GBN32_RS04760 | GBN32_RS04760 | -1.349745445 | 0.002100038 | 0.042364757 |
| GBN32_RS18205 | GBN32_RS18205 | 1.363673828 | 0.002291001 | 0.045911048 |
| GBN32_RS05100 | GBN32_RS05100 | -2.041721088 | 0.002322339 | 0.04623289 |
| GBN32_RS09710 | GBN32_RS09710 | 1.320007799 | 0.00234918 | 0.046461564 |
| GBN32_RS03100 | *hybO* | 1.335612599 | 0.002417707 | 0.047506371 |
| GBN32_RS02885 | GBN32_RS02885 | -6.519946129 | 0.002434933 | 0.047536173 |
| GBN32_RS04865 | GBN32_RS04865 | 1.346774279 | 0.002566788 | 0.04978911 |
| GBN32_RS09660 | *msrP* | 1.295775627 | 0.002737925 | 0.052770451 |
| GBN32_RS17065 | GBN32_RS17065 | 1.294676271 | 0.002780726 | 0.053256188 |
| GBN32_RS03775 | GBN32_RS03775 | 1.26542637 | 0.002808211 | 0.053444326 |
| GBN32_RS16650 | *uspE* | 1.242659884 | 0.002844454 | 0.053795731 |
| GBN32_RS11910 | GBN32_RS11910 | -1.331803037 | 0.002884602 | 0.054027794 |
| GBN32_RS08155 | *pomA* | -2.007066078 | 0.002892433 | 0.054027794 |
| GBN32_RS03090 | *hybB* | 1.308872311 | 0.002974351 | 0.055217099 |
| GBN32_RS17280 | GBN32_RS17280 | 1.451614663 | 0.003113205 | 0.057421553 |
| GBN32_RS04135 | GBN32_RS04135 | -1.577503151 | 0.00313105 | 0.057421553 |
| GBN32_RS16555 | *pnuC* | 1.50824167 | 0.003152169 | 0.057460619 |
| GBN32_RS17640 | *ppk2* | 1.305025565 | 0.003215452 | 0.058263221 |
| GBN32_RS02755 | GBN32_RS02755 | 1.24931872 | 0.003347368 | 0.060292474 |
| GBN32_RS05315 | GBN32_RS05315 | 1.774146614 | 0.003476403 | 0.06224613 |
| GBN32_RS12640 | GBN32_RS12640 | -2.93147276 | 0.003515158 | 0.062274489 |
| GBN32_RS06715 | GBN32_RS06715 | 1.234497184 | 0.003519147 | 0.062274489 |
| GBN32_RS07685 | GBN32_RS07685 | 1.262062746 | 0.003565404 | 0.062618667 |
| GBN32_RS07480 | *recD* | -1.356557745 | 0.003579983 | 0.062618667 |
| GBN32_RS17790 | GBN32_RS17790 | -1.279294075 | 0.003726771 | 0.06481155 |
| GBN32_RS09060 | GBN32_RS09060 | 2.398893108 | 0.003904919 | 0.067521622 |
| GBN32_RS11040 | *rpsQ* | -1.21587435 | 0.003955239 | 0.068003135 |
| GBN32_RS15120 | GBN32_RS15120 | -1.213197654 | 0.00412 | 0.07043571 |
| GBN32_RS02480 | *dmsD* | 1.295823595 | 0.00414731 | 0.070504262 |
| GBN32_RS02510 | *selB* | -1.252145286 | 0.004174781 | 0.070574784 |
| GBN32_RS02770 | *ybcJ* | 1.254778647 | 0.004345327 | 0.073049782 |
| GBN32_RS10345 | GBN32_RS10345 | 1.229110542 | 0.004428143 | 0.074030715 |
| GBN32_RS06420 | GBN32_RS06420 | -1.196696256 | 0.004472922 | 0.074368473 |
| GBN32_RS02910 | GBN32_RS02910 | 1.195746298 | 0.00451354 | 0.074417195 |
| GBN32_RS07140 | *erpA* | 1.210779455 | 0.004525038 | 0.074417195 |
| GBN32_RS08805 | *artM* | -1.196578166 | 0.004692249 | 0.076488569 |
| GBN32_RS00830 | *fdxH* | -1.183711662 | 0.004701545 | 0.076488569 |
| GBN32_RS09205 | *cysW* | -1.462218828 | 0.004746963 | 0.076814486 |
| GBN32_RS11270 | *ppc* | -1.189190702 | 0.004984213 | 0.080224622 |
| GBN32_RS05340 | GBN32_RS05340 | -1.633729066 | 0.005032069 | 0.080528384 |
| GBN32_RS14725 | GBN32_RS14725 | 1.255875851 | 0.00505631 | 0.080528384 |
| GBN32_RS16740 | *ycfP* | 1.24094741 | 0.00516462 | 0.081822725 |
| GBN32_RS17240 | GBN32_RS17240 | 1.194012032 | 0.005201591 | 0.081874468 |
| GBN32_RS05615 | *putP* | -1.166673034 | 0.0052322 | 0.081874468 |
| GBN32_RS11675 | *trmH* | -1.233866869 | 0.005249057 | 0.081874468 |
| GBN32_RS16075 | *purL* | -1.167423477 | 0.005302401 | 0.081954858 |
| GBN32_RS00810 | GBN32_RS00810 | 1.741161224 | 0.005308378 | 0.081954858 |
| GBN32_RS07495 | GBN32_RS07495 | 1.171061621 | 0.005356098 | 0.082271847 |
| GBN32_RS11935 | *hemC* | -1.172424455 | 0.005429352 | 0.082881106 |
| GBN32_RS02385 | *putA* | -1.156297931 | 0.005466654 | 0.082881106 |
| GBN32_RS09505 | *menE* | -1.253050865 | 0.005477932 | 0.082881106 |
| GBN32_RS05170 | *gspH* | -1.30588061 | 0.005537389 | 0.082959912 |
| GBN32_RS07345 | GBN32_RS07345 | 1.22053519 | 0.005537972 | 0.082959912 |
| GBN32_RS04255 | *pth* | 1.375891986 | 0.005570857 | 0.083041451 |
| GBN32_RS06735 | *petA* | -1.161039392 | 0.005614551 | 0.083282511 |
| GBN32_RS01800 | GBN32_RS01800 | 1.195829524 | 0.005885263 | 0.086872228 |
| GBN32_RS06335 | GBN32_RS06335 | 1.202457778 | 0.006037147 | 0.08860486 |
| GBN32_RS13070 | GBN32_RS13070 | -1.251436594 | 0.006061205 | 0.08860486 |
| GBN32_RS03825 | *mepA* | -1.234656549 | 0.006122515 | 0.089070822 |
| GBN32_RS01725 | GBN32_RS01725 | 1.391148716 | 0.006448024 | 0.09335752 |
| GBN32_RS01795 | GBN32_RS01795 | 1.425494206 | 0.00660823 | 0.095085405 |
| GBN32_RS15950 | *iscU* | 1.138054267 | 0.006630212 | 0.095085405 |
| GBN32_RS10550 | GBN32_RS10550 | 1.595057969 | 0.006686163 | 0.095435522 |
| GBN32_RS11285 | GBN32_RS11285 | -1.164553148 | 0.00673289 | 0.095651294 |
| GBN32_RS17030 | GBN32_RS17030 | 1.135009732 | 0.006879176 | 0.096693564 |
| GBN32_RS07015 | GBN32_RS07015 | 1.240813469 | 0.006940806 | 0.096693564 |
| GBN32_RS11890 | *yigB* | -1.431776366 | 0.006946155 | 0.096693564 |
| GBN32_RS08905 | GBN32_RS08905 | 1.320568859 | 0.006953612 | 0.096693564 |
| GBN32_RS05085 | *nfuA* | 1.132315033 | 0.006966027 | 0.096693564 |
| GBN32_RS12555 | *bcsB* | -1.127758586 | 0.007082161 | 0.097856711 |
| GBN32_RS11350 | *zapB* | 1.124796669 | 0.007254336 | 0.099507317 |
| GBN32_RS08855 | *hisD* | -1.546886592 | 0.007267388 | 0.099507317 |
| GBN32_RS15320 | GBN32_RS15320 | 1.140017101 | 0.007377415 | 0.100558814 |
| GBN32_RS07810 | *pdxJ* | 1.141577568 | 0.007430582 | 0.100829334 |
| GBN32_RS01625 | GBN32_RS01625 | 1.137641903 | 0.007493067 | 0.101223311 |
| GBN32_RS10750 | GBN32_RS10750 | 1.523104979 | 0.007787656 | 0.104585228 |
| GBN32_RS17275 | GBN32_RS17275 | 1.220277242 | 0.007811058 | 0.104585228 |
| GBN32_RS00900 | GBN32_RS00900 | 2.893543529 | 0.007864773 | 0.10484054 |
| GBN32_RS09065 | GBN32_RS09065 | 1.2577062 | 0.008027516 | 0.106496035 |
| GBN32_RS05630 | *hcp1* | -1.319011517 | 0.00808376 | 0.106496035 |
| GBN32_RS02890 | GBN32_RS02890 | 1.924123554 | 0.008135331 | 0.106496035 |
| GBN32_RS16065 | GBN32_RS16065 | 1.112289218 | 0.00816301 | 0.106496035 |
| GBN32_RS01740 | GBN32_RS01740 | 1.544162915 | 0.008232207 | 0.106496035 |
| GBN32_RS10455 | *tssE* | -1.105267176 | 0.008277065 | 0.106496035 |
| GBN32_RS07505 | GBN32_RS07505 | 1.170927084 | 0.008282242 | 0.106496035 |
| GBN32_RS11260 | *argC* | -1.119535421 | 0.008293839 | 0.106496035 |
| GBN32_RS09525 | *tyrP* | -1.16325318 | 0.008305705 | 0.106496035 |
| GBN32_RS03555 | *fliF* | 1.177262116 | 0.008645511 | 0.110385306 |
| GBN32_RS03540 | GBN32_RS03540 | 1.115490411 | 0.008712301 | 0.110770686 |
| GBN32_RS14860 | *thrA* | -1.136238836 | 0.008752619 | 0.110817674 |
| GBN32_RS00820 | *fdhE* | -1.130721114 | 0.0088392 | 0.111447577 |
| GBN32_RS17250 | *matP* | 1.187481468 | 0.008920998 | 0.112012199 |
| GBN32_RS11245 | *argH* | -1.095525178 | 0.009096555 | 0.113686756 |
| GBN32_RS16045 | *guaB* | 1.085534599 | 0.009129505 | 0.113686756 |
| GBN32_RS16375 | GBN32_RS16375 | 1.135821205 | 0.00931385 | 0.115507011 |
| GBN32_RS04370 | GBN32_RS04370 | -1.312964264 | 0.009522412 | 0.117492089 |
| GBN32_RS09735 | GBN32_RS09735 | -1.378579907 | 0.0095587 | 0.117492089 |
| GBN32_RS02450 | GBN32_RS02450 | -1.100402908 | 0.009686519 | 0.117492089 |
| GBN32_RS12695 | GBN32_RS12695 | -2.009862722 | 0.009732694 | 0.117492089 |
| GBN32_RS04770 | GBN32_RS04770 | -1.074118811 | 0.009743879 | 0.117492089 |
| GBN32_RS11465 | GBN32_RS11465 | -1.290198865 | 0.009761317 | 0.117492089 |
| GBN32_RS06810 | *murE* | -1.093022541 | 0.009790149 | 0.117492089 |
| GBN32_RS02495 | GBN32_RS02495 | 1.09180794 | 0.009844818 | 0.117492089 |
| GBN32_RS05095 | *bioH* | -1.347061203 | 0.009857465 | 0.117492089 |
| GBN32_RS04910 | *cydB* | 1.077410759 | 0.009862191 | 0.117492089 |
| GBN32_RS10380 | *nfsB* | 1.101441578 | 0.009904818 | 0.117537173 |
| GBN32_RS07350 | *ssrA* | 1.066231737 | 0.010131365 | 0.119578024 |
| GBN32_RS17205 | *nuoH* | -1.076540853 | 0.010155834 | 0.119578024 |
| GBN32_RS09670 | GBN32_RS09670 | 1.207875862 | 0.010288008 | 0.120590649 |
| GBN32_RS02820 | *nrfA* | 1.077817907 | 0.010321539 | 0.120590649 |
| GBN32_RS05150 | *gspD* | -1.105205594 | 0.010561022 | 0.122511353 |
| GBN32_RS05635 | GBN32_RS05635 | -1.377028578 | 0.010570774 | 0.122511353 |
| GBN32_RS08865 | GBN32_RS08865 | -1.111772155 | 0.01063925 | 0.122511353 |
| GBN32_RS15895 | GBN32_RS15895 | 1.064688555 | 0.01064788 | 0.122511353 |
| GBN32_RS12740 | *flgM* | 1.108733462 | 0.010893619 | 0.124863986 |
| GBN32_RS14760 | GBN32_RS14760 | 1.055021276 | 0.011057763 | 0.126267137 |
| GBN32_RS02790 | *rmuC* | 1.135975036 | 0.011161122 | 0.12669666 |
| GBN32_RS17580 | *bcp* | 1.071445019 | 0.011179117 | 0.12669666 |
| GBN32_RS08595 | *odhB* | -1.050224364 | 0.011358008 | 0.128243776 |
| GBN32_RS15400 | GBN32_RS15400 | 1.361445509 | 0.011442905 | 0.128722044 |
| GBN32_RS12560 | *bcsZ* | -1.118241296 | 0.011712651 | 0.130889805 |
| GBN32_RS05580 | *yjjG* | 1.071683564 | 0.011722121 | 0.130889805 |
| GBN32_RS16860 | GBN32_RS16860 | 1.156461299 | 0.011768568 | 0.130925318 |
| GBN32_RS16270 | *gloA* | 1.052967477 | 0.011839081 | 0.13101484 |
| GBN32_RS12570 | GBN32_RS12570 | -1.250797677 | 0.011863208 | 0.13101484 |
| GBN32_RS07625 | GBN32_RS07625 | -1.649040323 | 0.011908845 | 0.1310406 |
| GBN32_RS11130 | *mscL* | 1.106356882 | 0.012067315 | 0.132227441 |
| GBN32_RS00180 | GBN32_RS00180 | 1.44082753 | 0.012147795 | 0.132227441 |
| GBN32_RS09665 | GBN32_RS09665 | 1.44082753 | 0.012147795 | 0.132227441 |
| GBN32_RS13575 | *rsxD* | -1.06908099 | 0.012237988 | 0.132731731 |
| GBN32_RS17730 | GBN32_RS17730 | -1.100599097 | 0.012347763 | 0.13344404 |
| GBN32_RS02695 | GBN32_RS02695 | 1.114478592 | 0.012731599 | 0.136517431 |
| GBN32_RS14620 | *apaH* | -1.266735986 | 0.012760566 | 0.136517431 |
| GBN32_RS01080 | GBN32_RS01080 | -1.112793045 | 0.012803049 | 0.136517431 |
| GBN32_RS02835 | *nrfD* | 1.05282746 | 0.012813167 | 0.136517431 |
| GBN32_RS14745 | *leuA* | -1.061764058 | 0.012902258 | 0.136517431 |
| GBN32_RS02840 | *nrfE* | 1.128884366 | 0.012902837 | 0.136517431 |
| GBN32_RS14135 | GBN32_RS14135 | 1.265610112 | 0.013003216 | 0.137100109 |
| GBN32_RS16590 | GBN32_RS16590 | 1.060264255 | 0.013116445 | 0.137813761 |
| GBN32_RS03780 | GBN32_RS03780 | 1.49801784 | 0.013240194 | 0.138632615 |
| GBN32_RS04745 | *napB* | 1.208586691 | 0.013396387 | 0.139784369 |
| GBN32_RS08655 | GBN32_RS08655 | 1.036901316 | 0.014020024 | 0.145788983 |
| GBN32_RS07135 | *clcA* | -1.23077886 | 0.014194702 | 0.146657355 |
| GBN32_RS12180 | GBN32_RS12180 | -1.048646458 | 0.014200464 | 0.146657355 |
| GBN32_RS07200 | GBN32_RS07200 | -1.145302744 | 0.014314455 | 0.14733177 |
| GBN32_RS13195 | *thiH* | 1.543860596 | 0.01458372 | 0.14959436 |
| GBN32_RS09690 | GBN32_RS09690 | 1.319596302 | 0.014653994 | 0.149807383 |
| GBN32_RS12840 | dnaN | -1.034339796 | 0.014737788 | 0.149811357 |
| GBN32_RS08985 | GBN32_RS08985 | 1.19093367 | 0.014753399 | 0.149811357 |
| GBN32_RS08780 | *potG* | -1.051905795 | 0.014831719 | 0.150102947 |
| GBN32_RS14790 | GBN32_RS14790 | -1.561187851 | 0.014931034 | 0.150389849 |
| GBN32_RS04965 | GBN32_RS04965 | 1.153284038 | 0.014959466 | 0.150389849 |
| GBN32_RS05120 | GBN32_RS05120 | -1.043298428 | 0.015135509 | 0.151655794 |
| GBN32_RS12665 | *fliD* | 1.499287759 | 0.015229728 | 0.151812095 |
| GBN32_RS17605 | *wrbA* | -1.134316592 | 0.015251446 | 0.151812095 |
| GBN32_RS03965 | *folC* | -1.111683552 | 0.015393972 | 0.152728388 |
| GBN32_RS14915 | *tal* | 1.006135572 | 0.015501995 | 0.15327172 |
| GBN32_RS04495 | *cueR* | 1.235783622 | 0.015550039 | 0.15327172 |
| GBN32_RS06785 | *rnpB* | -1.001216558 | 0.015631007 | 0.15356957 |
| GBN32_RS07485 | *argA* | -1.014626356 | 0.016001586 | 0.15447244 |
| GBN32_RS14165 | GBN32_RS14165 | -1.019008399 | 0.016016648 | 0.15447244 |
| GBN32_RS08755 | *nfsA* | 1.054711778 | 0.016026755 | 0.15447244 |
| GBN32_RS06525 | *prmA* | -1.067891496 | 0.016057089 | 0.15447244 |
| GBN32_RS03235 | GBN32_RS03235 | 1.069871797 | 0.016062205 | 0.15447244 |
| GBN32_RS11915 | GBN32_RS11915 | -1.102244739 | 0.016080121 | 0.15447244 |
| GBN32_RS07330 | GBN32_RS07330 | 1.093670595 | 0.016080244 | 0.15447244 |
| GBN32_RS02765 | GBN32_RS02765 | 1.374123641 | 0.016452689 | 0.157550121 |
| GBN32_RS11315 | *priA* | -1.127336366 | 0.017067102 | 0.16214146 |
| GBN32_RS13865 | *mukE* | -1.025115467 | 0.017092959 | 0.16214146 |
| GBN32_RS01070 | GBN32_RS01070 | -1.058687776 | 0.017200456 | 0.16214146 |
| GBN32_RS08500 | *fur* | 1.001741047 | 0.017204949 | 0.16214146 |
| GBN32_RS15265 | GBN32_RS15265 | -1.879403226 | 0.018003167 | 0.168140688 |
| GBN32_RS08090 | GBN32_RS08090 | 1.479661023 | 0.018121122 | 0.168721588 |
| GBN32_RS09405 | *yfcE* | 1.033916341 | 0.018181411 | 0.168763652 |
| GBN32_RS07500 | GBN32_RS07500 | 1.03128083 | 0.0183078 | 0.168999248 |
| GBN32_RS00175 | GBN32_RS00175 | 1.139559337 | 0.018318491 | 0.168999248 |
| GBN32_RS11775 | *yihA* | 1.020181005 | 0.018396631 | 0.169204274 |
| GBN32_RS08795 | *rlmC* | -1.444805369 | 0.018502711 | 0.169664254 |
| GBN32_RS08160 | GBN32_RS08160 | -1.180445298 | 0.018592404 | 0.169793808 |
| GBN32_RS11905 | *dapF* | -1.023858375 | 0.018629063 | 0.169793808 |
| GBN32_RS06180 | *pyrH* | 1.001362991 | 0.018747304 | 0.170358382 |
| GBN32_RS05620 | GBN32_RS05620 | 1.002144337 | 0.018905013 | 0.171173737 |
| GBN32_RS15525 | GBN32_RS15525 | -1.227203643 | 0.018950166 | 0.171173737 |
| GBN32_RS16560 | GBN32_RS16560 | 1.338318475 | 0.019018094 | 0.171276052 |
| GBN32_RS13510 | *moaC* | -1.023954653 | 0.019702129 | 0.176386519 |
| GBN32_RS00890 | GBN32_RS00890 | 1.020063028 | 0.020136972 | 0.17974772 |
| GBN32_RS07930 | GBN32_RS07930 | 1.064632995 | 0.020487544 | 0.181417109 |
| GBN32_RS03695 | *iolD* | -1.036649815 | 0.020619424 | 0.181417109 |
| GBN32_RS02315 | GBN32_RS02315 | 1.626120179 | 0.020623756 | 0.181417109 |
| GBN32_RS09135 | GBN32_RS09135 | 1.204006313 | 0.021001375 | 0.183670982 |
| GBN32_RS17880 | GBN32_RS17880 | 3.326136089 | 0.021566548 | 0.186992479 |
| GBN32_RS06535 | GBN32_RS06535 | -3.149563561 | 0.021566548 | 0.186992479 |
| GBN32_RS13945 | GBN32_RS13945 | 3.326136089 | 0.021566548 | 0.186992479 |
| GBN32_RS17780 | GBN32_RS17780 | -1.587793266 | 0.02169529 | 0.187571282 |
| GBN32_RS09230 | *glnK* | 1.086307171 | 0.021917456 | 0.188952198 |
| GBN32_RS09625 | *yciH* | 1.039894022 | 0.022135215 | 0.190287387 |
| GBN32_RS06595 | GBN32_RS06595 | -1.015224477 | 0.022755017 | 0.191628721 |
| GBN32_RS10805 | GBN32_RS10805 | 1.182090953 | 0.022775533 | 0.191628721 |
| GBN32_RS02850 | GBN32_RS02850 | 1.011420541 | 0.023049899 | 0.192320669 |
| GBN32_RS08085 | GBN32_RS08085 | 2.130116679 | 0.023070854 | 0.192320669 |
| GBN32_RS10985 | GBN32_RS10985 | -1.201085164 | 0.023589839 | 0.194305297 |
| GBN32_RS17650 | *pstB* | 1.003495764 | 0.023621576 | 0.194305297 |
| GBN32_RS03895 | GBN32_RS03895 | 1.064171959 | 0.023869034 | 0.195188251 |
| GBN32_RS03575 | *fliJ* | 1.062722002 | 0.023980387 | 0.195188251 |
| GBN32_RS18160 | GBN32_RS18160 | -1.024341484 | 0.02428278 | 0.195764502 |
| GBN32_RS04390 | GBN32_RS04390 | 1.099955381 | 0.024326455 | 0.195764502 |
| GBN32_RS07475 | GBN32_RS07475 | -1.244505796 | 0.024389695 | 0.195764502 |
| GBN32_RS00425 | GBN32_RS00425 | 1.035262302 | 0.025095816 | 0.198795654 |
| GBN32_RS14675 | *thiQ* | -1.688617816 | 0.025475297 | 0.200750645 |
| GBN32_RS14695 | *rluA* | -1.068346943 | 0.026296473 | 0.20475243 |
| GBN32_RS03265 | GBN32_RS03265 | 1.008487103 | 0.026432821 | 0.20475243 |
| GBN32_RS11600 | GBN32_RS11600 | -1.709880968 | 0.026497687 | 0.20475243 |
| GBN32_RS01880 | GBN32_RS01880 | 1.097024328 | 0.026598839 | 0.20475243 |
| GBN32_RS12660 | *fliS* | -1.544893865 | 0.026831883 | 0.2055526 |
| GBN32_RS01655 | GBN32_RS01655 | -2.46602749 | 0.02704271 | 0.206644546 |
| GBN32_RS16490 | GBN32_RS16490 | 1.005447682 | 0.027233889 | 0.207581227 |
| GBN32_RS17105 | GBN32_RS17105 | -1.208216053 | 0.027314486 | 0.207672448 |
| GBN32_RS11290 | *metB* | -1.126222924 | 0.028088517 | 0.210586923 |
| GBN32_RS01090 | GBN32_RS01090 | -1.00084138 | 0.028918224 | 0.213654773 |
| GBN32_RS04165 | *nqrM* | 1.079770678 | 0.029038756 | 0.213654773 |
| GBN32_RS04150 | GBN32_RS04150 | 1.035766966 | 0.02908981 | 0.213654773 |
| GBN32_RS09410 | *yfcD* | 1.304872019 | 0.030298791 | 0.21938078 |
| GBN32_RS11645 | GBN32_RS11645 | -2.11257795 | 0.030348148 | 0.21938078 |
| GBN32_RS09865 | GBN32_RS09865 | 2.232721019 | 0.030348148 | 0.21938078 |
| GBN32_RS07120 | GBN32_RS07120 | -1.108330401 | 0.031014033 | 0.222648358 |
| GBN32_RS11595 | *crcB* | -1.31223038 | 0.031103696 | 0.222648358 |
| GBN32_RS03680 | GBN32_RS03680 | 1.022302232 | 0.03112368 | 0.222648358 |
| GBN32_RS18150 | GBN32_RS18150 | -1.37303937 | 0.031942348 | 0.225835385 |
| GBN32_RS12265 | *aqpZ* | -1.558103885 | 0.033533086 | 0.232731926 |
| GBN32_RS08750 | GBN32_RS08750 | 1.036594189 | 0.033619129 | 0.232773902 |
| GBN32_RS06575 | *mreD* | -1.173874713 | 0.034165251 | 0.234430952 |
| GBN32_RS15365 | *pspF* | -1.175389388 | 0.035303813 | 0.237927254 |
| GBN32_RS12210 | *cydB* | -1.175389388 | 0.035303813 | 0.237927254 |
| GBN32_RS01530 | GBN32_RS01530 | 1.275715954 | 0.036157187 | 0.240668153 |
| GBN32_RS00760 | GBN32_RS00760 | -1.058811798 | 0.03618771 | 0.240668153 |
| GBN32_RS07940 | GBN32_RS07940 | 1.025489667 | 0.038182333 | 0.250027019 |
| GBN32_RS10695 | GBN32_RS10695 | -1.408918123 | 0.040101413 | 0.257636679 |
| GBN32_RS12790 | *fliP* | -5.535581912 | 0.040964585 | 0.259871769 |
| GBN32_RS11990 | GBN32_RS11990 | 5.698633848 | 0.040964585 | 0.259871769 |
| GBN32_RS11625 | GBN32_RS11625 | 5.698633848 | 0.040964585 | 0.259871769 |
| GBN32_RS12775 | *fliE* | 5.698633848 | 0.040964585 | 0.259871769 |
| GBN32_RS00955 | GBN32_RS00955 | 1.396739159 | 0.041903977 | 0.261986435 |
| GBN32_RS08195 | GBN32_RS08195 | 1.162056008 | 0.042612006 | 0.264400947 |
| GBN32_RS05145 | *gspC* | -1.012224273 | 0.043338822 | 0.265532366 |
| GBN32_RS12505 | *yegD* | -1.803553704 | 0.045202178 | 0.27139244 |
| GBN32_RS10535 | GBN32_RS10535 | 1.938977097 | 0.045202178 | 0.27139244 |
| GBN32_RS14865 | GBN32_RS14865 | -1.295314707 | 0.046163285 | 0.27552288 |
| GBN32_RS09680 | GBN32_RS09680 | 1.198267166 | 0.048597149 | 0.285543634 |
| GBN32_RS00025 | GBN32_RS00025 | 1.198267166 | 0.048597149 | 0.285543634 |

Gene id：Gene number

log2FoldChange: The ratio of the gene expression levels of the treatment to control groups then taken as the logarithm of base 2

Pvalue: The p-value of the significance test

Padj: The p-values corrected for multiple hypothesis testing

**Table S1B**  Mass spectrometry protein detection results of *hcp* DNA-pull down (The following shows a part of the results)

| Protein | Description |  |
| --- | --- | --- |
| Lrp | Leucine-responsive transcriptional regulator |  |
| HexR | DNA-binding transcriptional regulator HexR |  |
| CysB | Transcriptional regulator CysB |  |
| CytR | LacI family transcriptional regulator |  |
| OmpR | Osmolarity response regulator |  |
| CsrA | Translational regulator CsrA |  |
| **RpoN** | **RNA polymerase sigma-54 factor** |  |
| ArcA | Two-component response regulator |  |
| OxyR | DNA-binding transcriptional regulator OxyR |  |
| IhfA | Integration host factor subunit alpha |  |
